# MRI Patterns Distinguish AQP4 Antibody Positive Neuromyelitis Optica Spectrum Disorder From Multiple Sclerosis

**DOI:** 10.3389/fneur.2021.722237

**Published:** 2021-09-09

**Authors:** Laura Clarke, Simon Arnett, Wajih Bukhari, Elham Khalilidehkordi, Sofia Jimenez Sanchez, Cullen O'Gorman, Jing Sun, Kerri M. Prain, Mark Woodhall, Roger Silvestrini, Christine S. Bundell, David A. Abernethy, Sandeep Bhuta, Stefan Blum, Mike Boggild, Karyn Boundy, Bruce J. Brew, Wallace Brownlee, Helmut Butzkueven, William M. Carroll, Cella Chen, Alan Coulthard, Russell C. Dale, Chandi Das, Marzena J. Fabis-Pedrini, David Gillis, Simon Hawke, Robert Heard, Andrew P. D. Henderson, Saman Heshmat, Suzanne Hodgkinson, Trevor J. Kilpatrick, John King, Christopher Kneebone, Andrew J. Kornberg, Jeannette Lechner-Scott, Ming-Wei Lin, Christopher Lynch, Richard A. L. Macdonell, Deborah F. Mason, Pamela A. McCombe, Jennifer Pereira, John D. Pollard, Sudarshini Ramanathan, Stephen W. Reddel, Cameron P. Shaw, Judith M. Spies, James Stankovich, Ian Sutton, Steve Vucic, Michael Walsh, Richard C. Wong, Eppie M. Yiu, Michael H. Barnett, Allan G. K. Kermode, Mark P. Marriott, John D. E. Parratt, Mark Slee, Bruce V. Taylor, Ernest Willoughby, Fabienne Brilot, Angela Vincent, Patrick Waters, Simon A. Broadley

**Affiliations:** ^1^Menzies Health Institute Queensland, Gold Coast, Griffith University, Southport, QLD, Australia; ^2^Department of Immunology, Pathology Queensland, Royal Brisbane and Women's Hospital, Herston, QLD, Australia; ^3^Nuffield Department of Clinical Neurosciences, John Radcliffe Hospital, University of Oxford, Oxford, United Kingdom; ^4^Department of Immunopathology, Westmead Hospital, Westmead, NSW, Australia; ^5^School of Pathology and Laboratory Medicine, University of Western Australia, Nedlands, WA, Australia; ^6^Department of Neurology, Wellington Hospital, Newtown, New Zealand; ^7^Department of Neurology, Princess Alexandra Hospital, Woolloongabba, QLD, Australia; ^8^Department of Neurology, Townsville Hospital, Douglas, QLD, Australia; ^9^Department of Neurology, College of Medicine and Public Health, Flinders University, Bedford Park, SA, Australia; ^10^Centre for Applied Medical Research, St. Vincent's Hospital, University of New South Wales, Darlinghurst, NSW, Australia; ^11^Department of Neurology, Auckland City Hospital, Grafton, New Zealand; ^12^Melbourne Brain Centre, Royal Melbourne Hospital, University of Melbourne, Parkville, VIC, Australia; ^13^Centre for Neuromuscular and Neurological Disorders, Queen Elizabeth II Medical Centre, Perron Institute for Neurological and Translational Science, University of Western Australia, Nedlands, WA, Australia; ^14^Department of Ophthalmology, Flinders Medical Centre, Flinders University, Bedford Park, SA, Australia; ^15^School of Medicine, Royal Brisbane and Women's Hospital, University of Queensland, Herston, QLD, Australia; ^16^Brain and Mind Centre, University of Sydney, Camperdown, NSW, Australia; ^17^Department of Neurology, Canberra Hospital, Garran, ACT, Australia; ^18^Sydney Medical School, Royal Prince Alfred Hospital, University of Sydney, Camperdown, NSW, Australia; ^19^Department of Neurology, Westmead Hospital, Westmead, NSW, Australia; ^20^South Western Sydney Medical School, Liverpool Hospital, University of New South Wales, Liverpool, NSW, Australia; ^21^Florey Institute of Neuroscience and Mental Health, University of Melbourne, Parkville, VIC, Australia; ^22^Department of Neurology, Royal Melbourne Hospital, Parkville, VIC, Australia; ^23^Department of Neurology, Royal Adelaide Hospital, Adelaide, SA, Australia; ^24^School of Paediatrics, Royal Children's Hospital, University of Melbourne, Parkville, VIC, Australia; ^25^Hunter Medical Research Institute, University of Newcastle, New Lambton Heights, NSW, Australia; ^26^School of Medicine, University of Auckland, Grafton, New Zealand; ^27^Department of Neurology, Austin Health, Heidelberg, VIC, Australia; ^28^Department of Neurology, Christchurch Hospital, Christchurch, New Zealand; ^29^Centre for Clinical Research, Royal Brisbane and Women's Hospital, University of Queensland, Herston, QLD, Australia; ^30^Neuroimmunology Group, Kids Neurosciences Centre, Children's Hospital at Westmead, University of Sydney, Westmead, NSW, Australia; ^31^Department of Neurology, Concord Repatriation General Hospital, Concord, NSW, Australia; ^32^School of Medicine, Deakin University, Waurn Ponds, VIC, Australia; ^33^Menzies Research Institute, University of Tasmania, Hobart, TAS, Australia; ^34^Department of Neurology, St. Vincent's Hospital, Darlinghurst, NSW, Australia; ^35^Department of Neurology, Gold Coast University Hospital, Southport, QLD, Australia

**Keywords:** neuromyelitis optica, multiple sclerosis, magnetic resonance imaging, diagnosis, NMOSD

## Abstract

Neuromyelitis optica spectrum disorder (NMOSD) and multiple sclerosis (MS) are inflammatory diseases of the CNS. Overlap in the clinical and MRI features of NMOSD and MS means that distinguishing these conditions can be difficult. With the aim of evaluating the diagnostic utility of MRI features in distinguishing NMOSD from MS, we have conducted a cross-sectional analysis of imaging data and developed predictive models to distinguish the two conditions. NMOSD and MS MRI lesions were identified and defined through a literature search. Aquaporin-4 (AQP4) antibody positive NMOSD cases and age- and sex-matched MS cases were collected. MRI of orbits, brain and spine were reported by at least two blinded reviewers. MRI brain or spine was available for 166/168 (99%) of cases. Longitudinally extensive (OR = 203), “bright spotty” (OR = 93.8), whole (axial; OR = 57.8) or gadolinium (Gd) enhancing (OR = 28.6) spinal cord lesions, bilateral (OR = 31.3) or Gd-enhancing (OR = 15.4) optic nerve lesions, and nucleus tractus solitarius (OR = 19.2), periaqueductal (OR = 16.8) or hypothalamic (OR = 7.2) brain lesions were associated with NMOSD. Ovoid (OR = 0.029), Dawson's fingers (OR = 0.031), pyramidal corpus callosum (OR = 0.058), periventricular (OR = 0.136), temporal lobe (OR = 0.137) and T1 black holes (OR = 0.154) brain lesions were associated with MS. A score-based algorithm and a decision tree determined by machine learning accurately predicted more than 85% of both diagnoses using first available imaging alone. We have confirmed NMOSD and MS specific MRI features and combined these in predictive models that can accurately identify more than 85% of cases as either AQP4 seropositive NMOSD or MS.

## Introduction

Neuromyelitis optica spectrum disorder (NMOSD) is an antibody-mediated autoimmune disease of the CNS in which the primary target for inflammation is aquaporin 4 (AQP4), a water channel found on the foot processes of astrocytes (1). NMOSD is frequently a disabling condition when untreated, with high relapse rates and a high risk of permanent neurological deficits following an attack (2). The treatment response in NMOSD is quite distinct to that seen in multiple sclerosis (MS) with NMOSD being highly steroid responsive and having a higher risk of disease recurrence on steroid withdrawal (3). Acute treatment with plasma exchange appears to be particularly effective in NMOSD (4). The risk of NMOSD relapses is diminished by immunosuppressive therapy, anti-B-cell therapies (e.g., rituximab, inebilizumab), anti-IL6 receptor and anti-complement component 5 monoclonal antibodies (5–8). Some immunomodulatory treatments for MS (β-interferon, fingolimod and natalizumab) may worsen NMOSD and alemtuzumab has shown no discernible benefit in small case series (9–11). Consequently, it is important to recognise NMOSD early and distinguish it from MS prior to commencing disease modifying therapy.

NMOSD and MS are inflammatory diseases of the CNS and lead to demyelination (12, 13). A considerable degree of overlap in MRI features has been noted (14). However, a number of groups have identified MRI features that are more common in NMOSD than in MS and may be useful in increasing the clinical suspicion for this diagnosis. Long lesions of the optic nerve (at least half the length of the optic nerve) and the spinal cord (at least 3 vertebral segments), and lesions of the area postrema have been incorporated into the current diagnostic criteria for seronegative NMOSD (1). Other MRI features, such as hypothalamic lesions, linear periventricular and brainstem periependymal lesions, and “bridging” lesions of the splenium have also been identified as having specificity for NMOSD when compared with MS (15–17). Some lesions typical for MS (e.g., perpendicular, ovoid, periventricular lesions, cerebellar peduncle lesions, juxtacortical lesions) appear to be less common in NMOSD (14, 18).

We report an observational, cross-sectional study of MRI characteristics in a cohort of AQP4 seropositive NMOSD cases collected across Australia and New Zealand compared with age and sex-matched MS cases from the same region. The aims of the study were to determine the clinical utility of specific MRI lesions and features in distinguishing NMOSD and MS, compare frequencies of specific lesions within cases and map the commonest locations of lesions in the spinal cord. We have gone on to develop an algorithm of MRI lesions and features to assist in distinguishing NMOSD from MS and applied this to the first sets of imaging available in this cohort. The hypotheses being tested were that (1) certain MRI features are associated with either NMOSD or MS (2) numbers of some lesions will be higher in MS than NMOSD, (3) spinal cord lesions will have predilection for different regions, and (4) that these lesions and features can be used to discriminate between these two conditions early in their clinical course, thereby expediting appropriate investigation and therapy.

## Materials and Methods

For this study we were only concerned with AQP4 antibody associated NMOSD and not MOG antibody associated demyelination (MOGAD). Firstly, lesions thought to be associated with NMOSD and MS were identified and defined from the prior literature. MRI from a large cohort of NMOSD and MS cases were then systematically reviewed and the presence and number of those lesions and features in both the “first” or “ever” imaging performed was determined. These data were then used to identify MRI lesions and features associated with NMOSD or MS. Lesion location in the spinal cord was also analysed. Finally, this association data was used to develop predictive algorithms to identify NMOSD and MS based purely on MRI findings.

### MRI Lesion and Feature Associations and Definitions

A literature review using the search terms NMOSD (and synonyms—neuromyelitis optica spectrum disorder, neuromyelitis optica, NMO, Devic's disease) and MRI (and synonyms—magnetic resonance imaging, MR imaging) for Title and Abstract fields was performed using MEDLINE and EMBASE databases on 17 January 2019. Searches were restricted to articles written in English. Potentially relevant articles looking at MRI features in NMOSD were identified from review of the title and abstract. These potential articles were then reviewed in full. Inclusion criteria were, (1) assessment of routine MRI lesions and features, (2) populations studied included NMOSD or limited forms of NMOSD (e.g., longitudinally extensive transverse myelitis) where relevant criteria for NMOSD or neuromyelitis optica were met (e.g., AQP4 antibody positive), and (3) original research or latest diagnostic criteria. The following exclusion criteria were applied (1) case reports with no relevant MRI findings, (2) no defined MRI findings or not relevant to NMOSD, (3) specialised MRI techniques or analysis, (4) review article or older diagnostic criteria, and (5) study solely focused on MOGAD. Additional articles were identified from reviewing the bibliographies of included articles and review articles. Articles relating to lesions associated with MS were identified from the 2010 update to the McDonald diagnostic criteria (19) and the literature supporting the MRI criteria contained therein. The available literature of the appearances of MRI lesions in NMOSD and MS were reviewed for evidence of specificity to either NMOSD or MS. However, it should be noted that our aim here was to be as inclusive as possible for any MRI lesion or feature that might be associated with NMOSD or MS. Consequently, there was no requirement for a statistically significant association. In addition, the same literature was reviewed to establish a clear definition for each lesion type that could be utilised in the review of MRI. Finally, for completeness the imaging sequence and anatomical counterparts for all lesions identified were added to the list and defined (e.g., corpus callosum Gd-enhancing lesion).

### NMOSD and MS Cohort Case Ascertainment

Potential NMOSD cases and MS cases were referred by 35 adult and paediatric neurologists across 23 clinical centres in Australia and New Zealand specialising in the assessment of patients with inflammatory diseases of the central nervous system as part of a prevalence survey as previously described (20, 21). For this study, NMOSD cases were defined according to the 2015 International panel for NMO diagnosis (IPND) criteria (1) with the additional requirement of being positive for AQP4 antibodies using either a tissue-based immunofluorescence technique or positivity on at least two cell-based assays (fixed, Euroimmun® or live, Oxford, UK) as previously described (22). Testing for MOG antibodies was completed on a subgroup of patients using either a fixed cell-based assay (Euroimmun®) or a fluorescence activated cell sorting live cell-based assay (Westmead Immunology, Sydney). Age and sex-matched MS cases were referred from each centre. The 2010 McDonald criteria (19) were used to confirm the diagnosis of MS. All MS cases were negative for AQP4 antibodies and had no clinical features suspicious for NMOSD. Basic demographic and clinical features were recorded for all cases as per a standardised data collection questionnaire as previously described (20). This study was approved by the Human Research Ethics Committees (HREC) of all participating centres (lead site HREC was Griffith University MED2009/646). All participants gave written informed consent to participate in this study.

### Review of MRI

MRI of the orbits, brain and spine were provided as raw DICOM images and stored on a secure server. Images were reviewed by at least two independent blinded reviewers (LC, SA, EK and SJS) using eFilm Workstation® 4.2.3, IBM Watson Health software on Eizo® RadiForce MX270W 68 cm monitors. In the case of disagreement, a third blinded expert reviewer (SBh) assessed the imaging and was the final arbiter. All blinded reviewers were trained in the identification of “typical” lesion patterns by the expert blinded reviewer.

Lesion numbers for each set of MRI on each subject were recorded using a bespoke Oracle® Database (Oracle Corporation®, Redwood Shores, California, US). The field strength, slice thickness and administration of gadolinium (Gd) contrast were recorded for each set of images. For analysis the first available imaging was counted separately (“first”) and then the maximum number of lesions of each type was also recorded across all subsequent sets of MRI for each patient (“ever”). The only exceptions to this were normal brain (not meeting Paty criteria), Paty criteria (23) and Barkhof criteria (24) which were only considered on the first imaging available. Lesions were then determined to be present (yes or no) on the “first” MRI or “ever” for any MRI on a “per patient” basis. Maximum numbers of each lesion type were also recorder per patient. MRI were defined as being related to a relapse if a documented relapse occurred either in the 90 days prior to the MRI or the 30 days following the MRI. All other MRI were deemed to have been undertaken during a period of remission.

**Figure 1:**
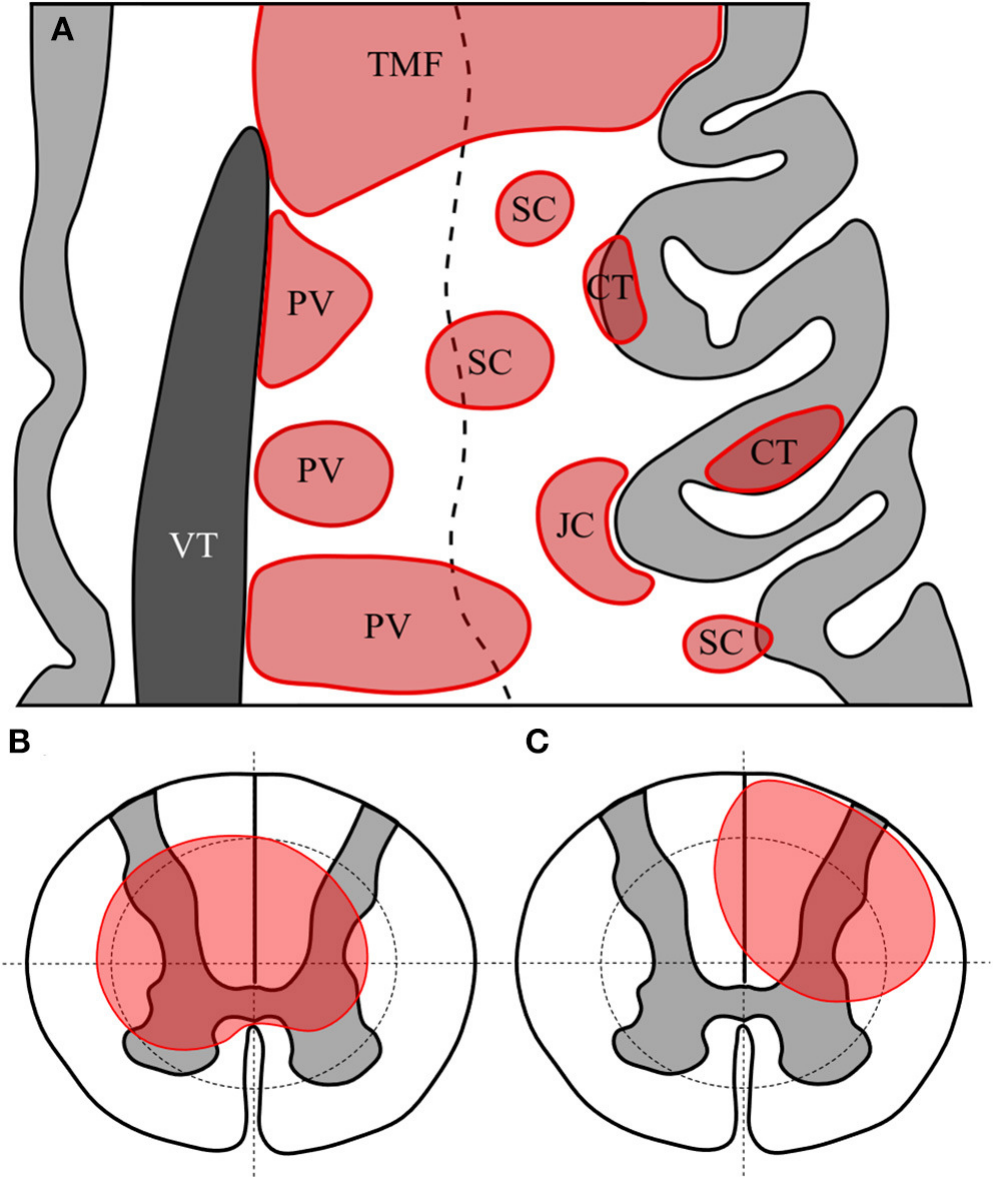
Representative axial section of left frontal cortex with cortex in light grey and lateral ventricle in dark grey **(A)**. White matter lesions are shown in red. TMF, tumefactive lesion—note size >3 cm and extension from ventricular surface to juxtacortical zone; PV, periventricular—note lesions abutting or immediately adjacent to lateral ventricle; CT, cortical—lesion wholly or predominantly located with the cortex; JC, juxtacortical—subcortical white matter lesion following the contour of the cortex but sparing the U-fibre layer; SC, subcortical—any other lesion predominantly located superficial to an imaginary line drawn half-way between the lateral ventricular surface and the cortex. Diagrammatic representation of axial section of spinal cord showing quadrantic segmentation of central and peripheral zones used to define lesions, showing **(B)** a central spinal cord lesion occupying at least a part of all central quadrants and **(C)** a partial spinal cord lesion occupying part of only three central regions.

Brain and optic nerve lesions were defined as specified in the prior literature. Conventions for distinguishing periventricular, subcortical, juxtacortical, cortical and tumefactive lesions are illustrated in Figure 1A. White matter lesions meeting criteria for more than one type of lesion that were not tumefactive were allocated according to the following preference order as appropriate: periventricular, cortical, juxtacortical then subcortical. Optic nerve lesions were recorded using standard brain MRI as well as dedicated images of the orbits where available.

Spinal cord lesions were defined and recorded as being in either the superior or inferior half of each vertebral level. This represented a total of 44 potential levels from the superior half of C1 down to the inferior half of L3, although no lesions were recorded at the L3 level as all spinal cords reviewed terminated at L2 or higher. Lesions in the spinal cord were determined to be “short” if they were <3 vertebral bodies (equivalent to <6 adjacent hemi-segmental levels) or “long” if they extended over 3 vertebral bodies or more (25). Where axial images were available spinal lesions were labelled as “central” if they involved all four quadrants of the centre of the spinal cord at a minimum of one level, or “partial” if they involved <4 quadrants of either the central or peripheral cord as illustrated in Figures 1B,C. Whole (axial) cord lesions were defined as involving all 8 central and peripheral quadrants on any one axial section. The presence of Gd-enhancement was noted for all spinal and brain lesions where contrast had been administered.

The distribution of spinal cord lesions has been illustrated using a “heat map” analogy (26). Long (≥3 vertebral segments) lesions, short lesions and Gd-enhancing lesions were colour coded and represented in columns for each subject with horizontal rows representing a vertebral half-segment. A summative approach using all spine MRI per patient was used. Summary frequencies of lesions at each hemi-vertebral level across all subjects were calculated on a proportional frequency basis.

### Statistical Analysis

Frequencies are expressed as *n*/*N* (%) and continuous data are presented as median (range) if not normally distributed or mean (standard deviation) if normally distributed. All analyses have been conducted on a per patient basis, thus “*N*” relates to the number of cases and not the number of scans. Comparisons of frequencies of different types of lesion between NMOSD and MS have been made using the “ever” data from all scans performed. Odds ratios and 95% confidence intervals were calculated using Haldane-Anscombe correction where frequencies for either NMOSD or MS were zero (27, 28). Odds ratios >1 favoured NMOSD and values <1 favoured MS. Results where the 95% confidence intervals did not cross 1 were taken as significant. No correction for multiple testing was applied for the following reasons: (1) there was prior evidence for association for many of the lesions and features assessed; (2) the NMOSD and MS cases were matched for age and sex, making adjustment for these baseline characteristics unnecessary; and (3) these were observational data (29). The relative value in distinguishing NMOSD from MS was assessed using sensitivity and specificity. Where imaging was not available the denominator was reduced. Lesion counts were assessed using appropriate non-parametric methods (Mann-Whitney *U*-test) (30). All analyses were performed on a per subject basis to not artificially inflate the “*N*” as might occur in a per MRI analysis. Statistical comparisons were performed using Statistical Package for Social Science (SPSS®) v25 (IBM® Chicago, US).

To create a predictive model for NMOSD and MS two approaches were undertaken utilising the “ever” MRI dataset. The first was an iterative approach in which MRI lesions and features associated with each condition were considered as present (1) or absent (0) and combined in either a summative (overall score) or regional (optic nerve, brain or spinal cord) subgrouping with variable weighting for each factor and region and variable cut-offs for each summative or regional score. Cut-offs were progressively increased using Excel®, Microsoft® (Seattle, US) until either a specificity of 1.00 was achieved or sensitivity fell below 0.50. Models with the highest specificity, aiming for a specificity >0.90, and highest sensitivity were then tested by sequentially removing features and increasing weightings and/or cut-offs for sub-scores until optimal values were achieved. The second approach used machine learning to create a predictive algorithm based on the same data using STATA® statistical software v14 (StataCorp® 2015, College Station, TX, USA). The utility of each model was assessed using weighted means (for both NMOSD and MS prediction) for the true positive rate (TPR), false positive rate (FPR), precision (positive predictive value), F-measure and receiver operator characteristic (ROC) area under the curve, with NMOSD as the positive state and MS as the negative state in the same “ever” data. Precision, F-measure and ROC area are all measures of accuracy and have a value from zero to one. The best performing models were then tested and compared with existing predictive models for MS (23, 31), NMOSD (14, 18) and NMOSD/MOGAD (32–34) (in the “first” MRI dataset). This was felt to most accurately reflect the clinical situation of a patient undergoing diagnostic evaluation. Again, weighted means for TPR, FPR, precision, F-measure and ROC area were used to asses diagnostic utility of the derived algorithms and prior methods. A sensitivity analysis including only MRI performed within 5 years of symptom onset was performed.

## Results

### NMOSD and MS MRI Lesion Identification and Definitions From Prior Literature

**Figure 2:**
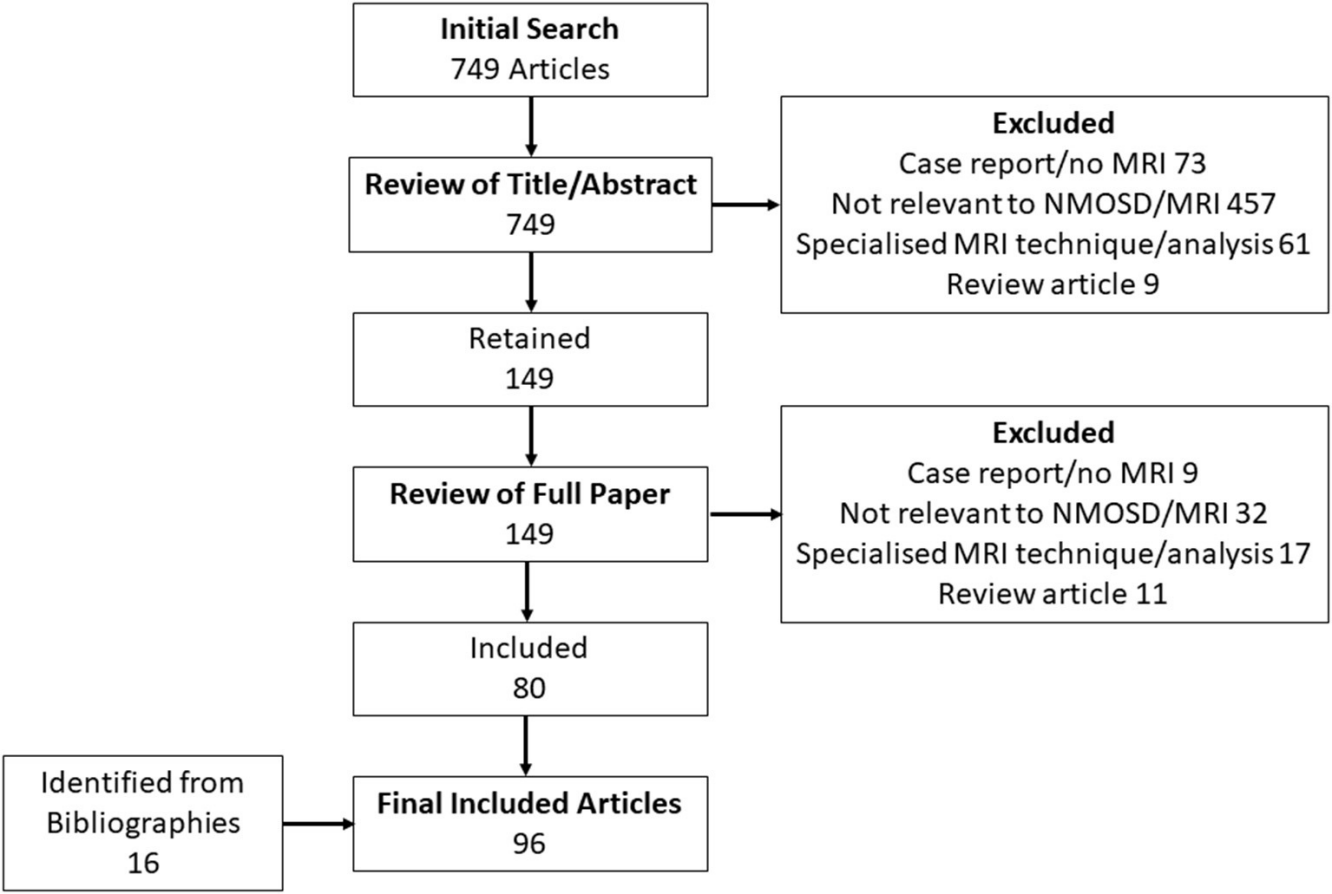
Flow chart of results of literature search used to identify MRI lesions and features associated with NMOSD and MS.

The initial literature search identified 426 potential articles relating to MRI features in NMOSD, of which 118 were selected for full review. These in turn yielded 71 included articles as summarised in Figure 2. A further five articles of relevance to NMOSD were identified from review of bibliographies of both the included articles and 10 review articles on MRI in NMOSD. Reasons for exclusion of articles are summarised in Figure 2. There were 11 articles of primary relevance to MS. A full list of articles reviewed and findings in brief is provided in Supplementary Table 1. Potential NMOSD (35) and MS (31) specific lesions together with their definitions and references are listed in Table 1. Thirty-four lesion types or MRI characteristics were identified as being potentially associated with NMOSD and 19 were potentially associated with MS. Some lesion types have been described in both MS and NMOSD. In this situation, lesions were either assigned the disease with greatest evidence for specificity in diagnosis (e.g., large supratentorial T2 lesions in MS) or were left neutral (e.g., cortical T2 lesions). Finally, natural anatomical counterparts to all lesion types or features were added and defined with no disease association as listed in Table 1. Most lesion types and definitions will be familiar to all, but two perhaps require further explanation. The first is whole (axial) cord lesion, which in this context refers to a cord lesion that involves the full axial section of the cord (i.e., all 8 central and peripheral quadrants of a single axial section as defined in Figures 1B,C). The second is “pyramidal” corpus callosum lesions which is a pyramidal shaped lesion arising from the inferior margin of the body of the corpus callosum, which we realised were the most commonly pictured callosal lesion in MS, although never really defined (18, 109).

**Table 1:** Identified MRI lesions and features with definitions and potential disease association.

**Region**	**Lesion/feature**	**Definition**	**Disease**	**References**
Optic pathway	Optic chiasm T2 or Gd	T2 hyperintense or Gd-enhancing lesion involving the optic chiasm.	NMOSD	(1, 36–41)
Optic pathway	Longitudinal optic nerve T2	T2 hyperintense or Gd-enhancing lesion of the optic nerve extending over at least 1/2 of the length of the optic nerve from the orbit to the chiasm.	NMOSD	(1, 39, 42, 43)
Optic pathway	Bilateral optic nerve T2 or Gd	T2 hyperintense or Gd-enhancing lesion of both optic nerves.	NMOSD	(39)
Brain	Normal brain	MR brain with no or few T2 hyperintense lesions, T1 black holes or Gd-enhancement and failing to meet Paty criteria.	NMOSD	(1, 44)
Brain	Leptomeningeal Gd	Gd-enhancement of the cerebral meninges.	NMOSD	(45, 46)
Supratentorial	Linear periventricular periependymal T2	T2 hyperintense lesion located immediately adjacent to and lying along the surface of the lateral ventricles, usually posteriorly (NOT perpendicular to lateral ventricles). Base of the lesion involves periependymal lining. Can be pencil thin and linear following ventricle lining, or thick and irregular.	NMOSD	(17, 18, 47–52)
Supratentorial	Punctate	T2 hyperintense “dot like” (<3 mm in diameter) lesion in the subcortical or deep white matter with discrete borders and does not abut on or lie perpendicular to the ventricles.	NMOSD	(17, 18, 48, 53)
Supratentorial	Patch	T2 hyperintense isolated lesion (<3 mm in diameter) in the subcortical or deep white matter with ill-defined borders and does not abut on or lie perpendicular to the ventricles.	NMOSD	(17, 48, 53)
Supratentorial	Cystic	T2 hyperintense white matter lesion that includes regions of cavitatory changes and/or an area of associated T1 hypointensity within the lesion.	NMOSD	(54)
Supratentorial	PRES-like	T2 hyperintense lesion usually involving both cortical and subcortical regions. Usually symmetrical and located in posterior, frontal, inferior temporal, cerebellar or brainstem regions. Must also demonstrate resolution over an interval of several months.	NMOSD	(55–57)
Supratentorial	Balo	Heterogenous lesion with alternating bands of demyelination and myelin preservation, often in whorl-like configurations. May also have bands of Gd-enhancement.	NMOSD	(58)
Supratentorial	Cloud-like	Multiple associated Gd-enhancing white matter lesions with patchy, heterogenous, often subtle, parenchymal enhancement with ill-defined/blurred margins on T1 weighted contrast imaging. Can be associated with periventricular periependymal enhancement (“flame” or “smoke coming out of the mouth” appearance).	NMOSD	(45, 59, 60)
Supratentorial	Tumefactive	Extensive and confluent T2 hyperintense, hemispheric white matter lesion which is >3 cm in its longest diameter and may have peripheral DWI restriction and Gd-enhancement on T1 contrast imaging (see Figure 1A).	NMOSD	(14, 17, 47, 49, 54, 60–63)
Supratentorial	Heterogeneous “acute” corpus callosum T2	Oedematous and heterogeneous T2 hyperintense lesion creating “marbled pattern” within corpus callosum.	NMOSD	(50, 64)
Supratentorial	Pencil-like corpus callosal T2 or Gd	Thin, linear hyperintense T2 lesion of the corpus callosum confined to the inferior periependymal layer.	NMOSD	(45)
Supratentorial	Bridging splenium T2	T2 hyperintense lesion forming an “arching bridge” across the splenium in axial views.	NMOSD	(47, 65)
Supratentorial	Third ventricle T2	T2 hyperintense lesion located adjacent to the third ventricle and involving parenchymal tissue (NOT isolated ependymal hyperintensity).	NMOSD	(17, 36, 40, 51, 61)
Supratentorial	Hypothalamic T2	T2 hyperintense lesion located in the hypothalamus and involving parenchymal tissue (NOT isolated ependymal hyperintensity). May have faint Gd-enhancement with poorly defined margins on T1 contrast sequence.	NMOSD	(16, 36, 40, 51, 66–68)
Supratentorial	Longitudinal corticospinal tract T2	T2 hyperintense lesion extending from the deep white matter through the posterior limb of the internal capsule to midbrain or pons. Can be unilateral or bilateral. Usually follows pyramidal tracts.	NMOSD	(17, 40, 49, 50, 69)
Infratentorial	Brainstem periependymal T2	Peripheral T2 hyperintense lesion of the brainstem involving the periependymal lining. Can be pencil thin and linear following ventricle lining, or thick and irregular.	NMOSD	(1, 18, 61)
Infratentorial	Anterior border midbrain T2	T2 hyperintense lesion involving the anterior border of the midbrain.	NMOSD	(36)
Infratentorial	Cerebral peduncle T2	T2 hyperintense lesion of the cerebral peduncle.	NMOSD	(14, 93)
Infratentorial	Periaqueductal T2	T2 hyperintense lesion located adjacent to the cerebral aqueduct and involving parenchymal tissue (NOT isolated ependymal hyperintensity).	NMOSD	(16, 36, 60, 61, 67)
Infratentorial	Floor of the fourth ventricle	Periependymal T2 hyperintense lesion involving the floor of the fourth ventricle.	NMOSD	(14, 17, 36, 45, 48, 50, 51, 60)
Infratentorial	Central medullary T2	T2 hyperintense lesion located in the central medulla and often extending into the adjacent upper cervical cord.	NMOSD	(17, 36, 49, 53, 60, 61, 67, 70–74)
Infratentorial	Nucleus tractus solitarius T2	T2 hyperintense lesion involving the nucleus tracts solitarius.	NMOSD	(75)
Infratentorial	Area postrema T2	T2 hyperintense lesion involving the area postrema.	NMOSD	(1, 40, 51, 61, 67, 76)
Spinal cord	Central T2 spinal cord	T2 hyperintense lesion of the spinal cord involving all 4 central quadrants of the cord for at least one hemi-vertebral level (see Figure 1B).	NMOSD	(16, 70, 71, 74, 77–87)
Spinal cord	Longitudinal T2 spinal cord	T2 hyperintense spinal cord lesion uniformly extending over 3 or more vertebral segments along the length of the cord.	NMOSD	(1, 16, 17, 51, 70, 72, 78, 80, 81, 83, 85, 88–96)
Spinal cord	Bright spotty T2 spinal cord	Small, rounded T2 hyperintense lesion of the spinal cord with high signal intensity similar to CSF.	NMOSD	(73, 74, 84, 97)
Spinal cord	Whole (axial) cord T2	T2 hyperintense lesion involving all 8 of the central and peripheral quadrants of the cord for at least one hemi-vertebral level (see Figure 1B).	NMOSD	(84, 86, 98)
Spinal cord	Cord swelling	T2 hyperintense lesion of the spinal cord associated with significant expansion of the spinal cord (>20% increase in midline sagittal diameter as compared with adjacent sections of spinal cord or loss of CSF space).	NMOSD	(74, 78, 87)
Spinal cord	Cord atrophy	Focal thinning of the spinal cord (more than 20% reduction in midline sagittal diameter as compared with adjacent sections of spinal cord) with or without myelomalacia.	NMOSD	(1, 48, 85, 87)
Spinal cord	Ring-enhancing T1	Rin-like circumferential Gd-enhancement of axial cross section of the spinal cord on T1 imaging	NMOSD	(78, 99)
Optic pathway	Optic nerve T2	T2 hyperintense lesion of the optic nerve.		
Optic pathway	Optic nerve Gd	Gd-enhancing lesion of the optic nerve.		
Brain	Brain Gd	Any Gd-enhancing lesion in the brain		
Supratentorial	Large supratentorial T2	Large (>6 mm), supratentorial T2 hyperintense lesion.		
Supratentorial	Cortical T2	T2 hyperintense lesion of the cortex (see Figure 1A).		
Supratentorial	Cortical Gd	Gd-enhancing lesion of the cortex.		
Supratentorial	Periventricular Gd	Gd-enhancing lesion of the periventricular region.		
Supratentorial	Subcortical T2	T2 hyperintense white matter lesion which is >3 mm but <3 cm in diameter and is not periventricular or juxtacortical (see Figure 1A).		
Supratentorial	Subcortical Gd	Gd-enhancing subcortical lesion.		
Supratentorial	Juxtacortical Gd	Gd-enhancing lesion of the juxtacortical region.		
Supratentorial	Corpus callosum T2	Any T2 hyperintense lesion confined to the parenchymal corpus callosum (NOT ependymal hyperintensity alone).		
Supratentorial	Rounded corpus callosum T2	Round, soft edged T2 lesion contained within wholly within the body of the corpus callosum (similar to the “snowball” lesion of Susac's syndrome).		
Supratentorial	Other corpus callosum T2	Any other corpus callosum lesion not described elsewhere.		
Supratentorial	Corpus callosum Gd	Gd-enhancing lesion of the corpus callosum.		
Supratentorial	Splenium T2	T2 hyperintense lesions located in the splenium at the posterior portion of the corpus callosum (NOT bridging lesions).		
Supratentorial	Deep Grey Matter T2	T2 hyperintense lesion of the basal ganglia (excluding thalamus and hypothalamus).		
Supratentorial	Thalamic T2	T2 hyperintense lesion located in the thalamus.		
Supratentorial	Deep Grey Matter Gd	Gd-enhancing lesion of the basal ganglia (excluding thalamus and hypothalamus).		
Supratentorial	Hypothalamic Gd	Gd-enhancing lesion of the hypothalamic parenchymal tissue.		
Supratentorial	Temporal lobe T2	Any T2 lesion within the temporal lobe.		
Infratentorial	Large infratentorial T2	Large (>6 mm) infratentorial T2 hyperintense lesion		
Infratentorial	Brainstem Gd	Gd-enhancing lesion of the brainstem.		
Infratentorial	Cerebellar Gd	Gd-enhancing lesion of the cerebellum (NOT involving the cerebellar peduncles).		
Infratentorial	Cerebellar peduncle Gd	Gd-enhancing lesion of the cerebellar peduncle.		
Spinal cord	Spinal cord Gd	Gd-enhancing lesion of the spinal cord on T1 contrast imaging.		
Brain	Paty criteria	MR brain with a minimum of 3 or more white matter lesions >3 mm in diameter or 2 white matter lesions if one of them is periventricular.	MS	(23, 100)
Brain	Barkhof criteria	MR brain where at least three of the following criteria are met: (1) Gd-enhancing lesion or 9 or more T2 lesions; (2) 1 infratentorial lesion; (3) 1 juxtacortical lesion; and (4) 3 periventricular lesions.	MS	(24, 101–104)
Brain	Large T2	T2 hyperintense lesion measuring >6 mm in longest diameter (can be supratentorial or infratentorial).	MS	(24, 105)
Brain	New T2	MR brain with 1 or more new T2 lesions compared to last available scan.	MS	(19, 31)
Brain	New Gd	MR brain with 1 or more new Gd-enhancing lesions compared to last available scan with contrast.	MS	(31)
Supratentorial	Black hole	T1 hypointense lesion of the white matter	MS	(87)
Brain	Nine or more T2	Nine or more hyperintense T2 lesions in the brain.	MS	(102)
Supratentorial	Periventricular T2	T2 hyperintense lesion adjacent to and abutting margins of the ventricles (see Figure 1A).	MS	(14, 19, 24, 31–34, 39, 44, 101, 102, 105, 106)
Supratentorial	Ovoid T2	Clearly circumscribed, elliptical T2 hyperintense lesion orientated perpendicular to the lateral ventricles.	MS	(24, 44, 49, 62)
Supratentorial	Dawson's fingers	Two or more periventricular T2 white matter lesions extending perpendicularly superiorly towards, but not necessarily reaching the juxtacortical zone.	MS	(14, 33, 34)
Supratentorial	Juxtacortical T2	T2 hyperintense lesion adjacent to and following the cortex and involving the cortical U-fibre layer (see Figure 1A).	MS	(14, 19, 24, 31–34, 39, 44, 87, 101, 102)
Supratentorial	Pyramidal corpus callosum T2	Pyramidal T2 hyperintense lesion of the corpus callosum arising from the inferior (periependymal) margin.	MS	(18, 87)
Supratentorial	Inferior Temporal Lobe T2	T2 hyperintense lesion of the inferior temporal lobe, bounded superiorly by the lateral ventricle and posteriorly by a line drawn between the pre-occipital notch and the inferior border of the splenium.	MS	(14, 33, 34)
Infratentorial	Infratentorial T2	T2 hyperintense lesion involving the infratentorial region of the brain (brainstem or cerebellum).	MS	(19, 31, 105)
Infratentorial	Brainstem T2	T2 hyperintense lesion of the brainstem (NOT including the following: anterior midbrain, cerebral peduncle, cerebral aqueduct, brainstem periependymal, area postrema, nucleus tractus solitarius and central medulla).	MS	(18, 24, 94, 101, 102)
Infratentorial	Cerebellar peduncle T2	T2 hyperintense lesion of the cerebellar peduncle.	MS	(24, 101, 102)
Infratentorial	Cerebellar T2	T2 hyperintense lesion of the cerebellum (NOT involving the cerebellar peduncles).	MS	(24, 101, 102)
Spinal cord	Short T2 spinal cord	T2 hyperintense lesion of the spinal cord extending for <3 vertebral segments.	MS	(19, 31, 81, 106, 107)
Spinal cord	Partial T2 spinal cord	T2 hyperintense lesion of the spinal cord involving fewer than 4 central or peripheral quadrants of the cord (see Figure 1C).	MS	(81, 85, 103, 106–108)

*NMOSD, neuromyelitis optica spectrum disorder; MS, multiple sclerosis*.

Reference list for Supplementary Table 1 and Table 1 (1, 14, 16–19, 23, 24, 31–34, 36–103, 105–108, 110–121).

### NMOSD and MS Cohorts and MRI Availability

There were 67 NMOSD cases who met 2015 IPND criteria, had full clinical data and were positive for AQP4 antibodies. There were 101 MS cases who met the 2010 McDonald criteria, had full clinical data, had no features suspicious for NMOSD, and were negative for AQP4 antibodies. MRI of orbits, brain or spine was available for 66/67 (99%) of NMOSD cases and 100/101 (99%) of MS cases (99% MRI availability overall). Consequently, one NMOSD case and one MS case were excluded from further analysis. Basic demographic and clinical features of the included cases are given in Table 2. These data indicate that NMOSD and MS cases were well-matched for age and sex but differed with regard to clinical features in a pattern that has been previously identified for these two disorders (104, 122, 123). All 66 included NMOSD cases were positive for AQP4 antibodies [38/66 (58%) using a cell-based assay and 28/66 (42%) using tissue immunofluorescence]. All 100 included MS cases were negative for AQP4 antibodies with 52/100 (52%) being tested on a cell-based assay. In our previously published analysis, the specificity of our tissue-based indirect immunofluorescence assays was 99.7% (95% CI 98.4–99.9%) (22). In addition, 48/66 (73%) of NMOSD cases and 52/100 (52%) of MS cases were tested for MOG antibodies and all were negative (22).

**Table 2:** Clinical features of NMOSD cases and MS controls.

**Clinical feature**	**NMOSD**	**MS**	***p*-value**
*N*	67	100	
Age (years)–median (range)	49 (19–85)	46 (16–73)	ns
Sex (Female)–*n*/*N* (%)	60/67 (90)	85/100 (85)	ns
Age at Onset (Years)–median (range)	41 (13–85)	32 (6–59)	<0.001
Disease Duration (Years)–median (range)	3.8 (0.1–43.1)	12.1 (0.5–43.4)	<0.001
Relapses–median (range)	4 (1–16)	3 (0–11)	ns
Annualised relapse rate–mean (SD)	0.78 (0.17–3.33)	0.33 (0.06–3.78)	<0.001
EDSS–median (range)	4 (0–9)	2 (0–9)	<0.001
Clinical Course–*n* (%)			0.016^*^
Monophasic (CIS)	9 (13)	12 (12)	
Relapsing remitting	56 (84)	73 (73)	
Secondary progressive	2 (3)	13 (13)	
Primary progressive	0 (0)	2 (2)	
CSF protein elevation–*n*/*N* (%)	19/42 (45)	3/39 (8)	<0.001
CSF white cell count elevation–*n*/*N* (%)	18/35 (51)	4/36 (11)	<0.001
Local synthesis of OCB–*n*/*N* (%)	8/42 (19)	29/40 (73)	<0.001

*NMOSD, neuromyelitis optica spectrum disorders; MS, multiple sclerosis; SD, standard deviation; EDSS, expanded disability status scale; CIS, clinically isolated syndrome; CSF, cerebrospinal fluid; OCB, oligoclonal bands*.

* *Overall comparison of all four groups*.

**Figure 3:**
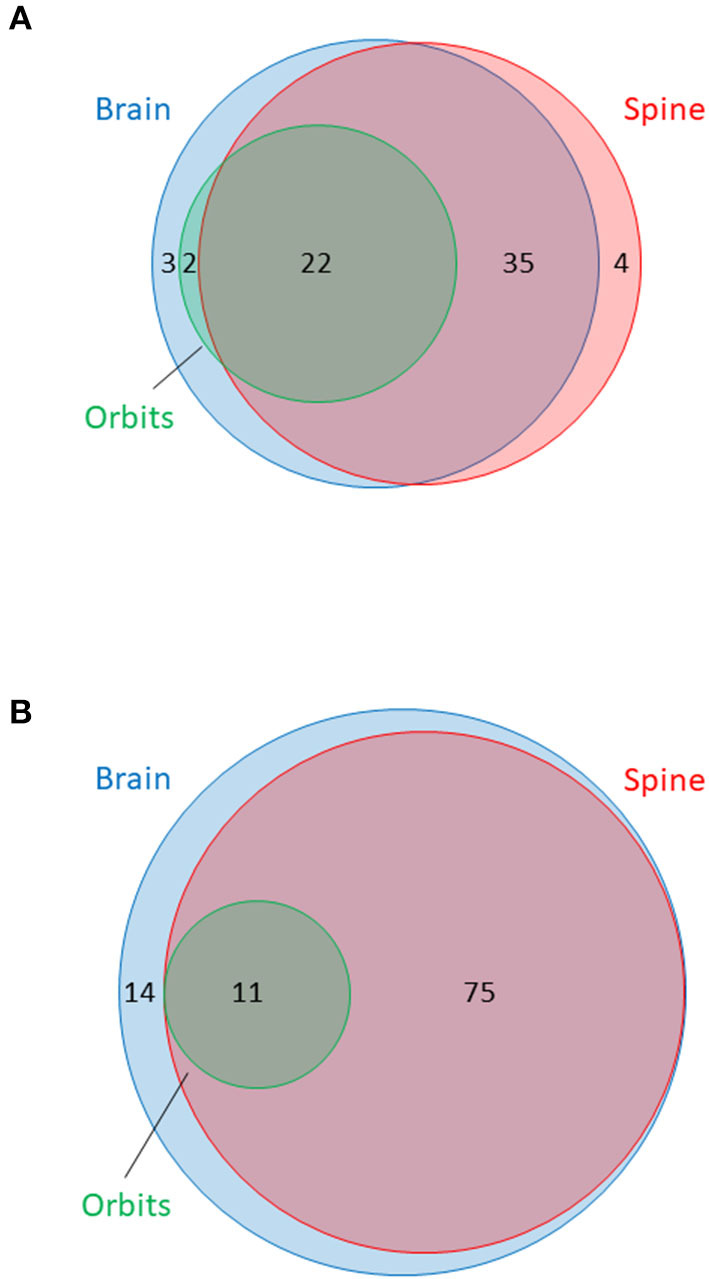
Venn diagram summarising availability of MRI of brain, orbits and spine in **(A)** NMOSD cases and **(B)** multiple sclerosis cases. Surface area of circles is proportional to the total number of MRI of each type. MRI brain in blue, orbits in green and spine in red. Numbers indicate numbers of patients with at least one MRI of specified type within each category or overlapping group.

The availability of MRI is summarised in Table 3. In total, 733 sets of MRI were reviewed. MRI brain was available for 62/66 (94%) and MRI spine was available for 61/66 (92%) of NMOSD cases. MRI brain was available for 100/100 (100%) and MRI spine was available for 86/100 (86%) of MS cases. Dedicated MRI of the orbits was more frequently available in NMOSD than in MS cases [18/66 (27%) of NMOSD cases vs. 6/100 (6%) of MS cases, *P* < 0.001]. MRI in NMOSD were more likely to have been obtained during a relapse (50% for brain and 49% for spine MRI in NMOSD vs. 13% for brain and 16% for spine MRI in MS; *p* < 0.0001). The availability of MRI per patient in NMOSD and MS is shown in Figure 3. The median time to first imaging from first symptoms was 9 months for brain and 12 months for spine MRI in the NMOSD cohort. The equivalent times for MS were both 10 years reflecting the greater disease duration of these cases from a historical cohort. Many MS cases had onset prior to 2000 and it was not possible to obtain DICOM files for MRI performed prior to the early 2000's as these were generally not centrally stored prior to then.

**Table 3:** Total numbers and types of MRI reviewed.

**MR Imaging**	**NMOSD**	**MS**	**Total**	***p*-value**
Cases–*N*	66	100	166	
**MRI Brain (scans)–** ***N***	**139**	**298**	**437**	
Scans per case–median (range)	1 (0–10)	2 (1–10)		
**Magnet–** ***n*** **(%)**				
0.5/1T	1 (1)	1 (0)		
1.5T	100 (72)	207 (70)	309 (71)	ns
3T	37 (27)	90 (30)	127 (29)	
**Axial slice thickness–** ***n*** **(%)**				
<5 mm	53 (38)	121 (41)	174 (40)	ns
5 mm or greater	86 (62)	177 (59)	263 (60)	
**Axial sequence–** ***n*** **(%)**				
FLAIR	124 (89)	265 (90)	389 (89)	ns
T2	15 (11)	33 (10)	48 (11)	
**Gd given–** ***n*** **(%)**				
Yes	89 (64)	172 (58)	261 (60)	ns
No	50 (36)	126 (42)	176 (40)	
**Timing–** ***n*** **(%)**				
During relapse	70 (50)	40 (13)	110 (25)	<0.0001
In remission	69 (50)	258 (87)	327 (75)	
Orbital Imaging–*n* (%)	43 (31)	12 (4)	27 (6)	<0.0001
**MRI Spine (scans)-N**	**134**	**166**	**300**	
Scans per case–median (range)	1.5 (0–8)	1 (0–8)		
**Magnet–** ***n*** **(%)**				
1.5T	95 (71)	135 (81)	230 (77)	0.034
3T	39 (29)	31 (19)	70 (23)	
**Sagittal slice thickness–** ***n*** **(%)**				
<4 mm	79 (59)	115 (69)	194 (65)	ns
4 mm or greater	55 (41)	51 (31)	106 (35)	
**Gd given–** ***n*** **(%)**				
Yes	83 (62)	78 (47)	161 (540)	0.01
No	51 (38)	88 (53)	139 (46)	
**Timing–** ***n*** **(%)**				
During relapse	66 (49)	27 (16)	93 (31)	<0.0001
In remission	68 (51)	139 (84)	207 (69)	
**Total Scans**	**273**	**464**	**737**	

*MRI, magnetic resonance imaging; NMOSD, neuromyelitis optica spectrum disorders; MS, multiple sclerosis; T, Tesla; FLAIR, T2 weighted Fluid Attenuated Inversion Recovery; T2, T2 weighted image; Gd, gadolinium*.

### MRI Features of NMOSD and MS

**Figure 4:**
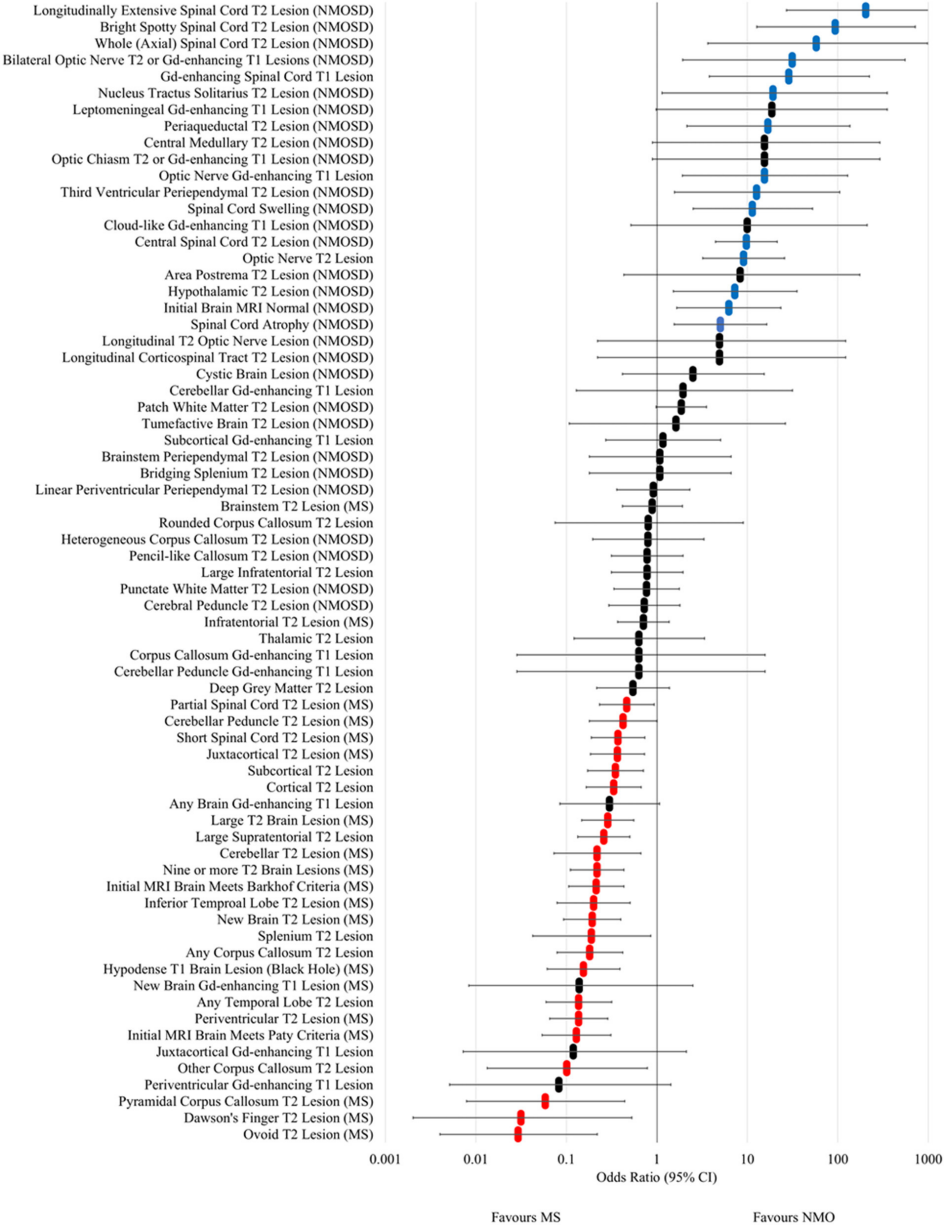
Forest plot of odds ratios for lesion occurrence in NMOSD and multiple sclerosis. OR >1 favour NMOSD and OR <1 favour multiple sclerosis. Error bars indicate 95% confidence intervals. Lesions significantly associated with NMOSD are highlighted in blue and those associated with multiple sclerosis are highlighted in red.

**Figure 5:**
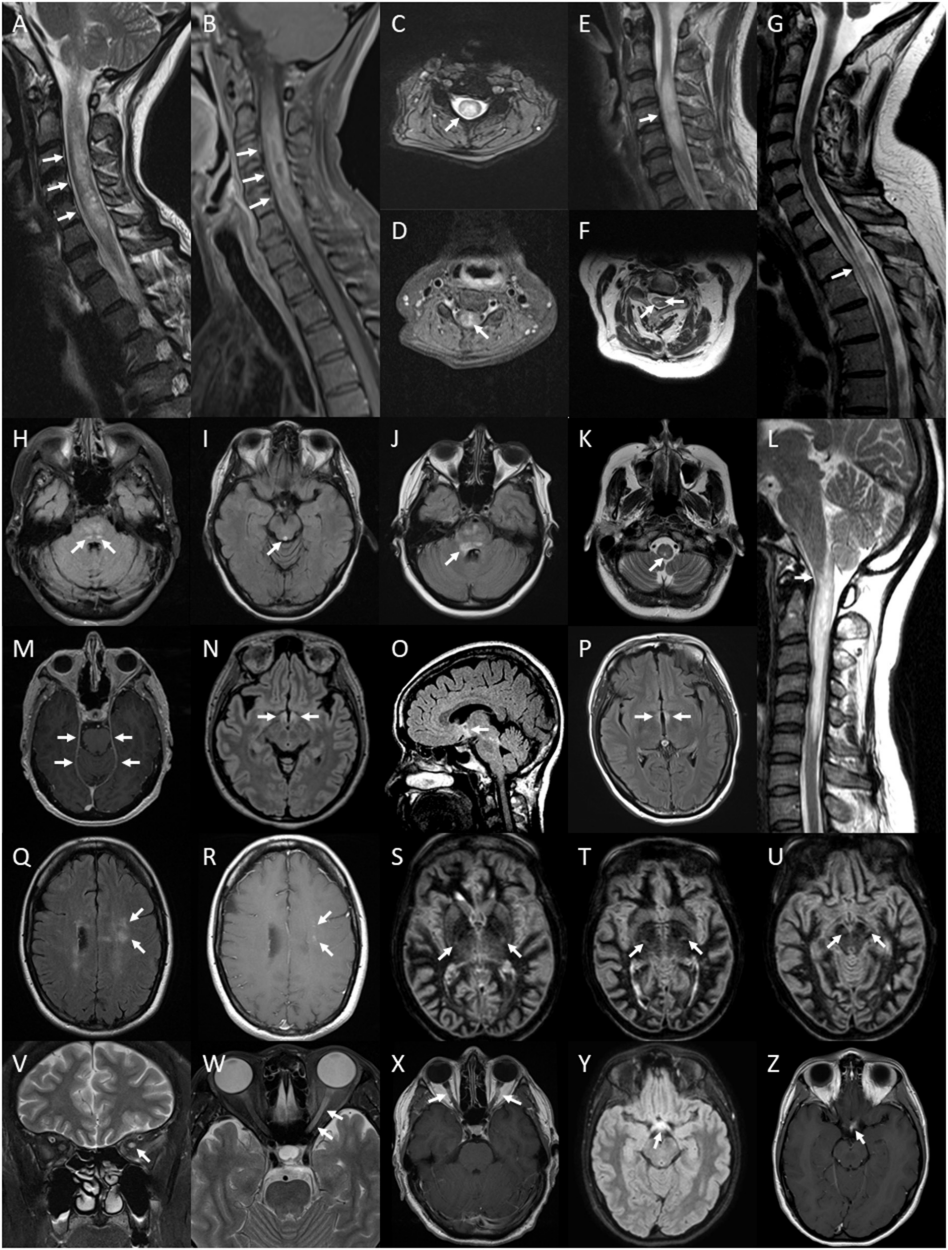
Lesions of the spinal cord, brain and optic nerve associated with or exclusively seen in NMOSD. Spinal cord lesions: longitudinally extensive spinal cord lesion (arrows) seen on T2 sagittal image of the cervical cord **(A)**, peripheral Gd-enhancing lesion (arrows) seen on T1 sagittal image of the same lesion **(B)**, central cord lesion (arrow) on T2 axial image at the level of C4 from the same lesion **(C)** and central Gd-enhancement (arrow) on T1 axial image in the same region **(D)**; swelling (arrow) of a high signal lesion on T2 sagittal image of the cervical region **(E)**; bright spotty cord lesions (arrows) on axialT2 image of the cervical region **(F)**; spinal cord atrophy with myelomalacia (arrow) on sagittal T2 image of the cervico-thoracic region **(G)**; Brainstem lesions: bilateral nucleus tractus solitarius high signal lesions (arrows) on axial FLAIR image through the pons **(H)**; periaqueductal high signal lesion (arrow) on axial FLAIR image through the midbrain **(I)**; high signal FLAIR lesion involving the floor of the fourth ventricle on axial FLAIR imaging at the level of the pons **(J)**; central medullary lesion (arrow) on T2 axial image of the medulla **(K)** and sagittal T2 image of high cervical cord lesion showing extension into the medulla **(L)**. Leptomeningeal enhancement of the tent (arrows) on axial gadolinium enhanced T1 image at the level of the midbrain **(M)**. Brain FLAIR lesions: hypothalamic high signal lesion (arrows) on axial image **(N)** and midline sagittal image **(O)**; high signal lesion involving the walls of the third ventricle (arrows) on axial image **(P)**. Cloud-like Gd-enhancing lesions (arrows) shown on axial FLAIR image **(Q)** and Gd-enhanced T1 image **(R)**. Bilateral longitudinally extensive cortico-spinal tract lesions (arrows) seen on sequential axial DIR images through the basal ganglia and midbrain **(S–U)**. Optic nerve lesions: high signal lesion of the left optic nerve (arrow) on coronal T2 image of the orbits **(V)**; longitudinally extensive high signal lesion of the left optic nerve (arrows) on axial T2 image of the orbits **(W)**; bilaterally Gd-enhancing lesions of the optic nerves (arrows) on axial Gd-enhanced T1 image of the orbits **(X)**; optic chiasm lesion (arrow) on axial Flair image **(Y)** and Gd-enhanced T1 image **(Z)**.

The odds ratios for occurrence in NMOSD vs. MS at any time for MRI lesion types and features are shown in Figure 4 and Supplementary Table 2. Longitudinally extensive spinal cord T2, bright spotty cord T2, whole (axial) cord T2, bilateral optic nerve T2/Gd-enhancing, Gd-enhancing spinal cord T1, nucleus tractus solitarius T2, periaqueductal T2, Gd-enhancing optic nerve T1, third ventricular periependymal T2, spinal cord swelling, central spinal cord T2, optic nerve T2, hypothalamic T2, initial brain MRI normal and spinal cord atrophy lesions and features were found to be associated with NMOSD. In addition, leptomeningeal Gd-enhancing T1, central medullary T2, optic chiasm T2 or Gd-enhancing T1, cloud-like Gd-enhancing T1, area postrema T2, longitudinal T2 optic nerve and longitudinal corticospinal tract T2 lesions had odds ratios that crossed one, but were only seen in NMOSD cases and not in MS. Examples of each of these NMOSD lesions is illustrated in Figure 5. NMOSD spinal cord lesions and features were seen at some time point (“ever”) in 45/62 (73%) of NMOSD cases. NMOSD brain lesions were seen in 23/62 (37%) and optic nerve lesions in 25/62 (40%) of NMOSD cases at some point in time.

**Figure 6:**
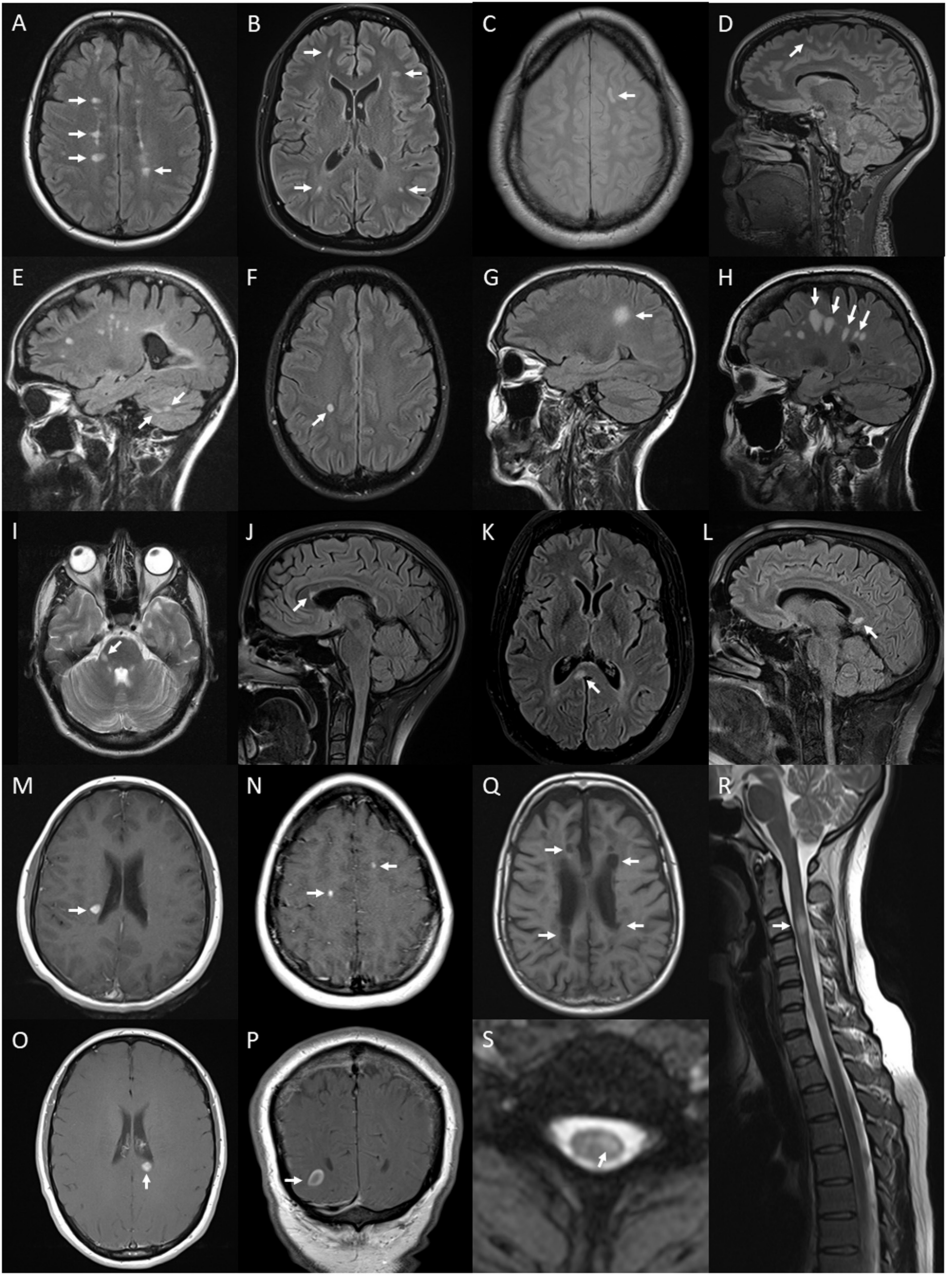
Lesions and features on MRI of brain and spinal cord associated with multiple sclerosis: periventricular hyperintense T2 white matter lesions (arrows) on axial FLAIR image of the brain **(A)**; subcortical T2 white matter lesions (arrows) on axial FLAIR image of the brain **(B)**; juxtacortical hyperintense T2 white matter lesion (arrow) on axial proton density image of the brain **(C)**; cortical hyperintense T2 lesion (arrow) on sagittal FLAIR image of the brain **(D)**; cerebellar hyperintense T2 lesions (arrows) on sagittal FLAIR image of the brain **(E)**; ovoid hyperintense T2 periventricular lesion (arrow) on axial FLAIR image of the brain **(F)**; large supratentorial T2 lesion (arrow) on sagittal FLAIR image of the brain **(G)**; Dawson's finger lesions (arrows) on sagittal FLAIR image of the brain **(H)**; right cerebellar peduncle hyperintense T2 lesion (arrow) on axial T2 image of the brain **(I)**; pyramidal corpus callosum hyperintense T2 lesion (arrow) on sagittal FLAIR image of the brain **(J)**; splenium hyperintense T2 lesion (arrow) on axial **(K)** and sagittal **(L)** FLAIR images of the brain; Gd-enhancing T1 lesions of periventricular (arrow) **(M)**, juxtacortical (arrows) **(N)**, splenium (arrow) **(O)** regions on axial T1 images of the brain and ring-enhancing lesion (arrow) on coronal T1 image of the brain **(P)**; hypointense T1 (black hole) lesions (arrows) on axial T1 image of the brain **(Q)**; short segment C3 T2 lesion (arrow) on sagittal T2 image of the cervical spinal cord **(R)**; and hyperintense T2 partial cord lesion (arrow) on axial T2 image of the cervical spinal cord **(S)**.

In MS, ovoid T2, Dawson's finger, pyramidal corpus callosum T2, other corpus callosum T2, initial MRI brain meeting Paty criteria, periventricular T2, any temporal lobe T2, hypodense T1 brain (black hole), any corpus callosum T2, splenium (non-bridging) T2, new T2 brain, inferior temporal lobe T2, initial MRI brain meeting Barkhof criteria, nine or more T2 brain, cerebellar T2, large supratentorial T2, large T2 brain, cortical T2, subcortical T2, juxtacortical T2, and cerebellar peduncle T2 lesions in the brain were associated with MS. Short segment and partial spinal cord T2 lesions were also associated with MS. Periventricular, juxtacortical and subcortical Gd-enhancing T1 lesions were not individually associated with MS but when combined (“any” brain Gd-enhancing lesion) trended towards being associated (OR 0.30; 95% CI 0.08–1.07) and when cloud-like Gd-enhancing lesions were excluded, this association became significant (OR 0.10; 95% CI 0.01–0.74). Examples of the above MS associated lesions are illustrated in Figure 6.

**Figure 7:**
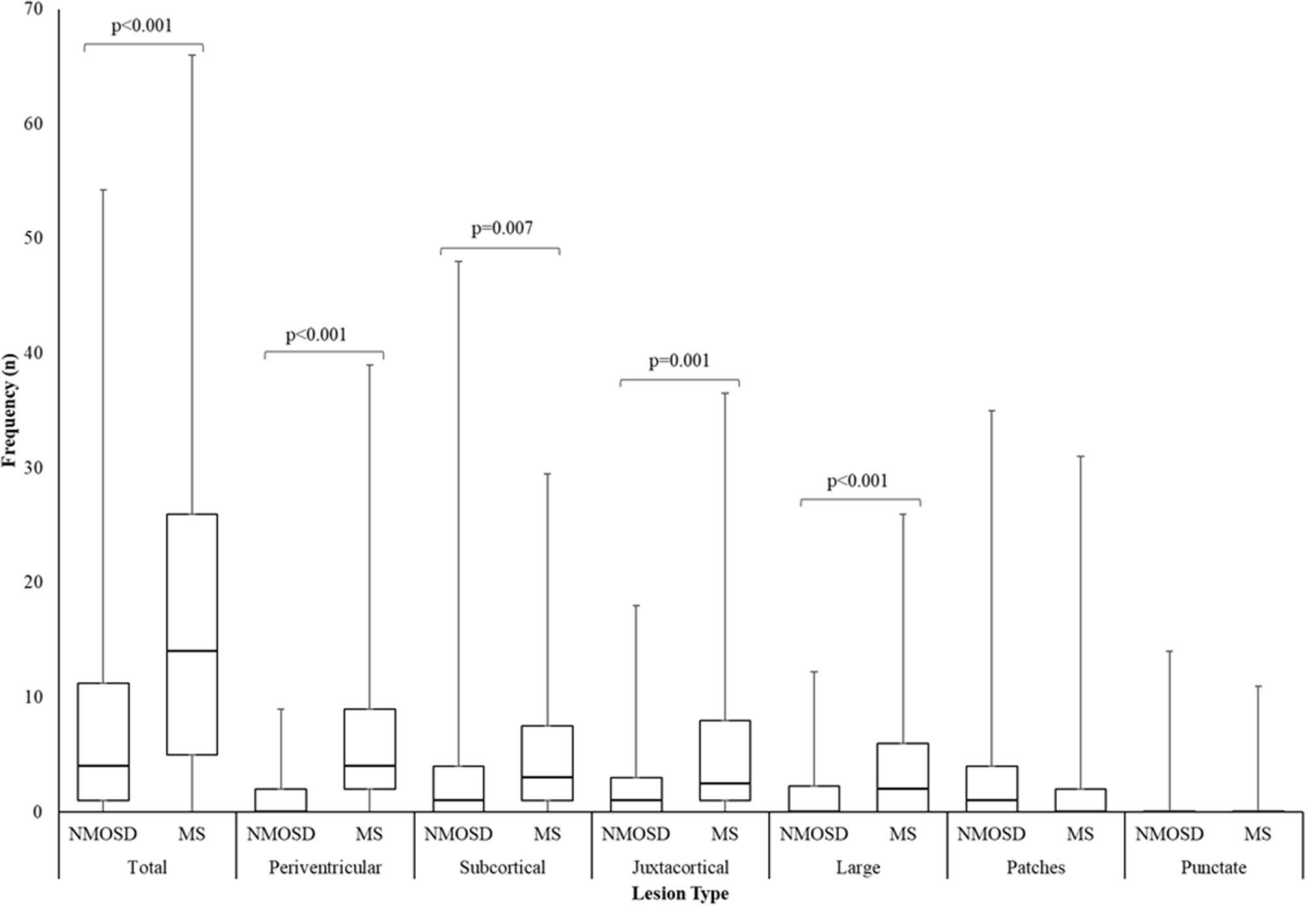
Box and whisker plot of the number of T2 lesions seen in NMOSD and multiple sclerosis for the most numerous brain lesion types. Lesion counts were the highest number of unique lesions seen on an individual scan from all MRI per patient. Central bar indicates median, boxes show interquartile range and whiskers show range.

**Figure 8:**
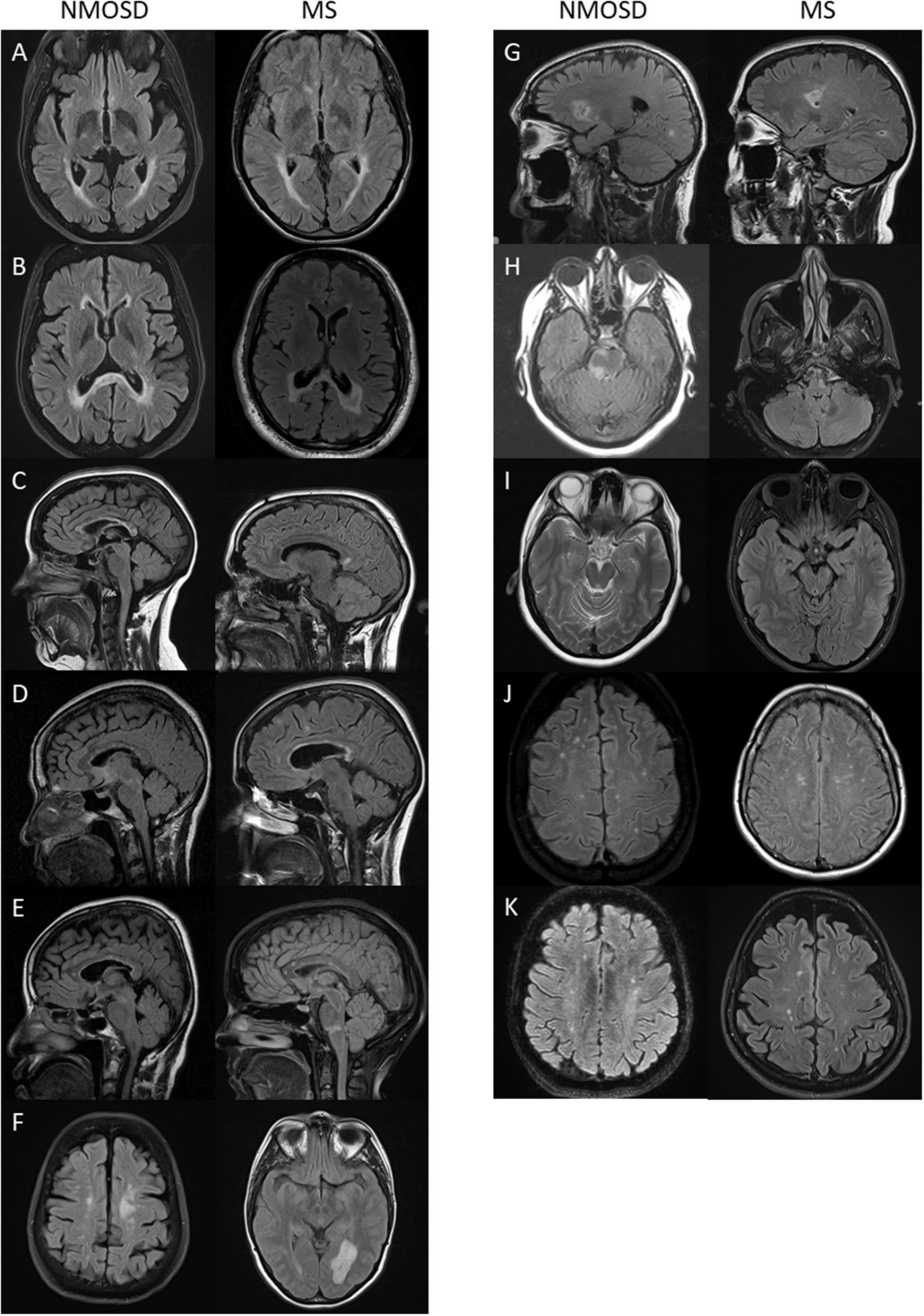
Lesions previously described in NMOSD that were seen with equal frequency in NMOSD (left panel) and multiple sclerosis (right panel): **(A)** linear periventricular periependymal T2 lesions; **(B)** “bridging” T2 lesion of the splenium; **(C)** heterogenous T2 lesion of the corpus callosum; **(D)** rounded corpus callosum lesion; **(E)** pencil-like corpus callosum lesion; **(F)** tumefactive white matter lesion; **(G)** cystic brain lesion; **(H)** periependymal brainstem T2 lesion; **(I)** cerebral peduncle lesion (here seen bilaterally in multiple sclerosis); **(J)** punctate white matter lesions; and **(K)** patch white matter lesions.

The commonest types of T2 lesion seen (periventricular, subcortical, juxtacortical and large T2) and total T2 lesion counts were more numerous in MS than NMOSD as shown in Figure 7. The median (range) total number of T2 lesions seen on any MRI brain were 4 (0–47) for NMOSD and 14 (0–54) for MS (*p* < 0.001; Mann-Whitney *U*-test). There were no differences in the frequencies of patch and punctate white matter lesions in NMOSD and MS (Figure 7). The following lesions previously noted in NMOSD: linear periventricular periependymal, bridging splenium, brainstem periependymal, cystic, heterogeneous corpus callosum, cerebral peduncle, punctate and patch lesions, were all found with similar frequencies in both NMOSD and MS (Figure 4; Supplementary Table 2). Examples of these lesions from NMOSD and MS cases are illustrated in Figure 8.

No Gd-enhancing T1 lesions of the cortex, corpus callosum, basal ganglia, hypothalamic region or brainstem were seen in NMOSD or MS cases. In addition, no anterior midbrain, posterior reversible encephalopathy syndrome-like, Balo-like, floor of fourth ventricle T2 or ring-enhancing Gd-enhancement of the spinal cord lesions were seen.

### Spinal Cord Lesion Location

**Figure 9:**
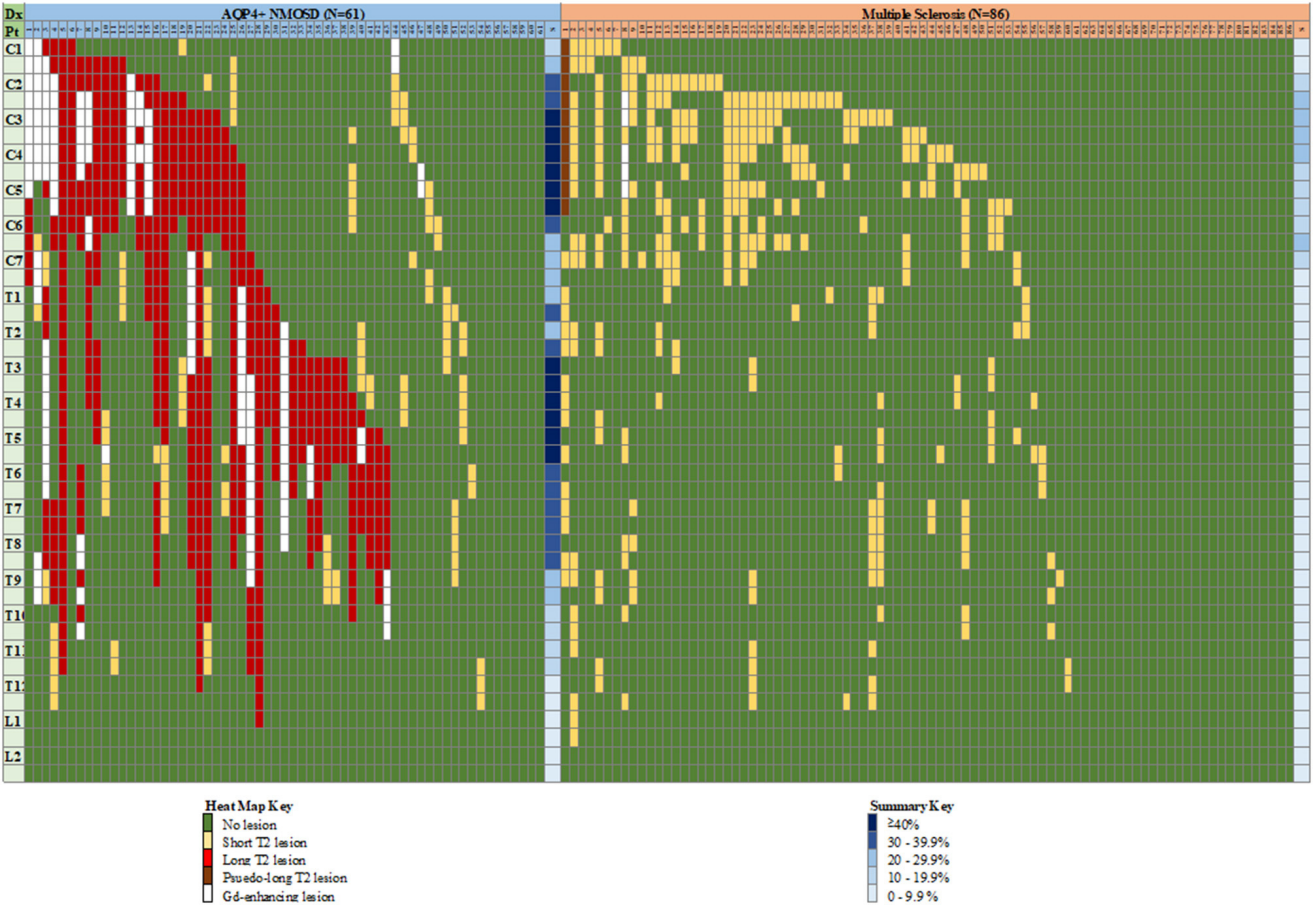
“Heat map” of lesion location in the spinal cord of individual cases with NMOSD and multiple sclerosis. Vertical columns indicate individual patients as numbered, horizontal rows indicate hemi-vertebral distances (i.e., two rows = one vertebral segment). Representation of lesions is for lesions appearing at any time during the disease course for an individual patient, hence consecutive short lesions spanning more than 3 vertebrae can be seen (i.e., multiple short lesions at different times). See text for definition of pseudo-long T2 lesion. Columns at far right of each group indicate a summary (S) of relative frequency of lesions for each level (any type of lesion). Dx, diagnosis; Pt, patient; C, cervical; T, thoracic; L, lumbar; NMOSD, neuromyelitis optica spectrum disorder; S, summary.

**Figure 10:**
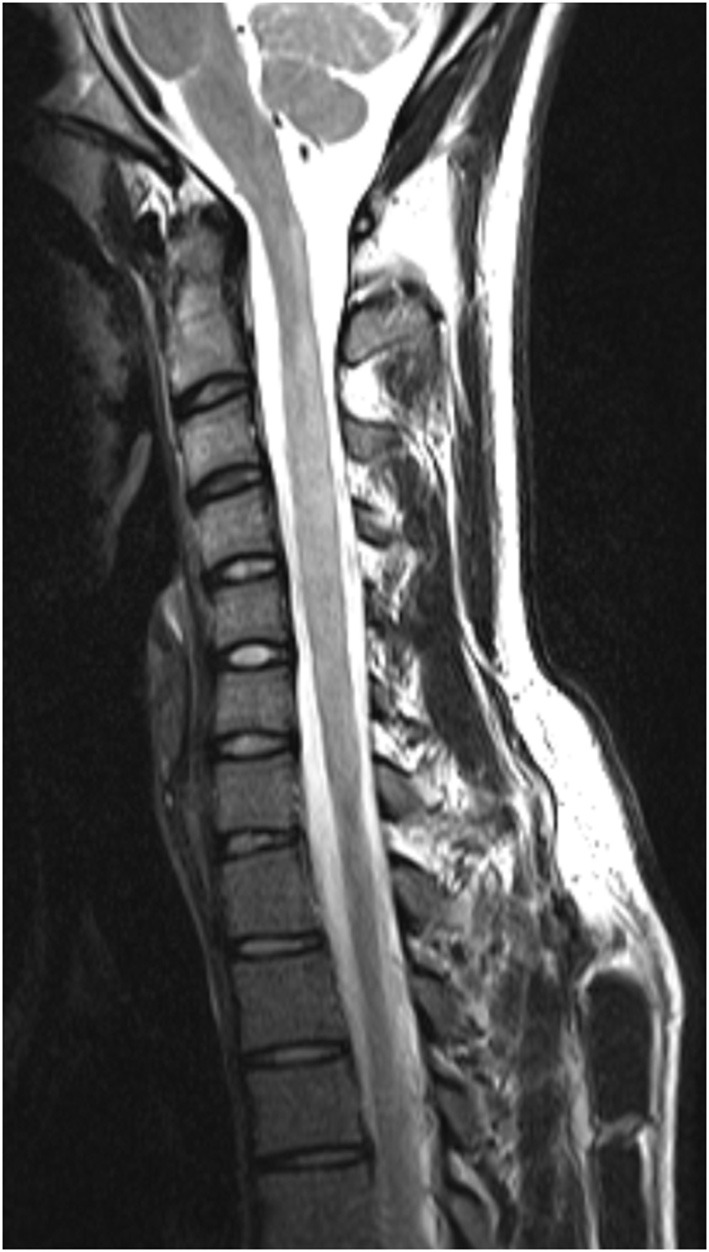
“Pseudo-longitudinally” extensive spinal cord lesion seen in multiple sclerosis. Sagittal T2 MRI of the cervical spine in a multiple sclerosis control showing longitudinally extensive high signal changes extending from C1 to C6. This lesion is confluent over at least 3 vertebral segments. Prior imaging confirmed that this lesion arose as a confluence of smaller lesions over time.

The distribution of spinal cord lesions at any stage of disease for each subject are summarised in Figure 9 as a “heat map.” A longitudinally extensive spinal cord lesion was seen in one MS case in the cervical region (Figure 10). It was only through review of prior imaging that it was clear that this lesion had arisen over time due to a coalescence of smaller lesions rather than a single focus of inflammation but remained in our count of longitudinally extensive spinal cord lesions as the appearance met our definition. Gd-enhancement was only evident in the spinal cords of 1/85 (1%) MS cases. The predominant site for cord lesions in MS was the cervical region, with lesions between C2 and C7 being seen in 10–30% of cases. MRI spine was persistently normal in 26/86 (30%) of MS cases. In contrast, longitudinally extensive spinal cord lesions were seen in 43/61 (70%) and Gd-enhancement was evident in 19/61 (31%) of NMOSD cases. No spinal cord lesions were evident in 7/61 (11%) of NMOSD cases. The most common location for spinal cord lesions in NMOSD were the cervical (C2–C6) and mid-thoracic regions (T2–T8) where lesions at each hemi-vertebral level were present in more than 30% of cases.

### Diagnostic Utility of MRI Features

The relative frequencies, sensitivities, specificities and odds ratios for associated MRI features in the diagnosis of NMOSD and MS are given in Supplementary Table 2. It is notable that most of the MRI features associated with NMOSD have high specificity but low sensitivity, except for longitudinally extensive spinal cord lesions. In diagnosing MS, ovoid, periventricular, pyramidal corpus callosum, other corpus callosum, splenium, cerebellar and T1 black hole lesions have high specificity with low sensitivity, whilst periventricular lesions have high sensitivity and modest specificity.

### Predictive Modelling Based on MRI Features

**Figure 11:**
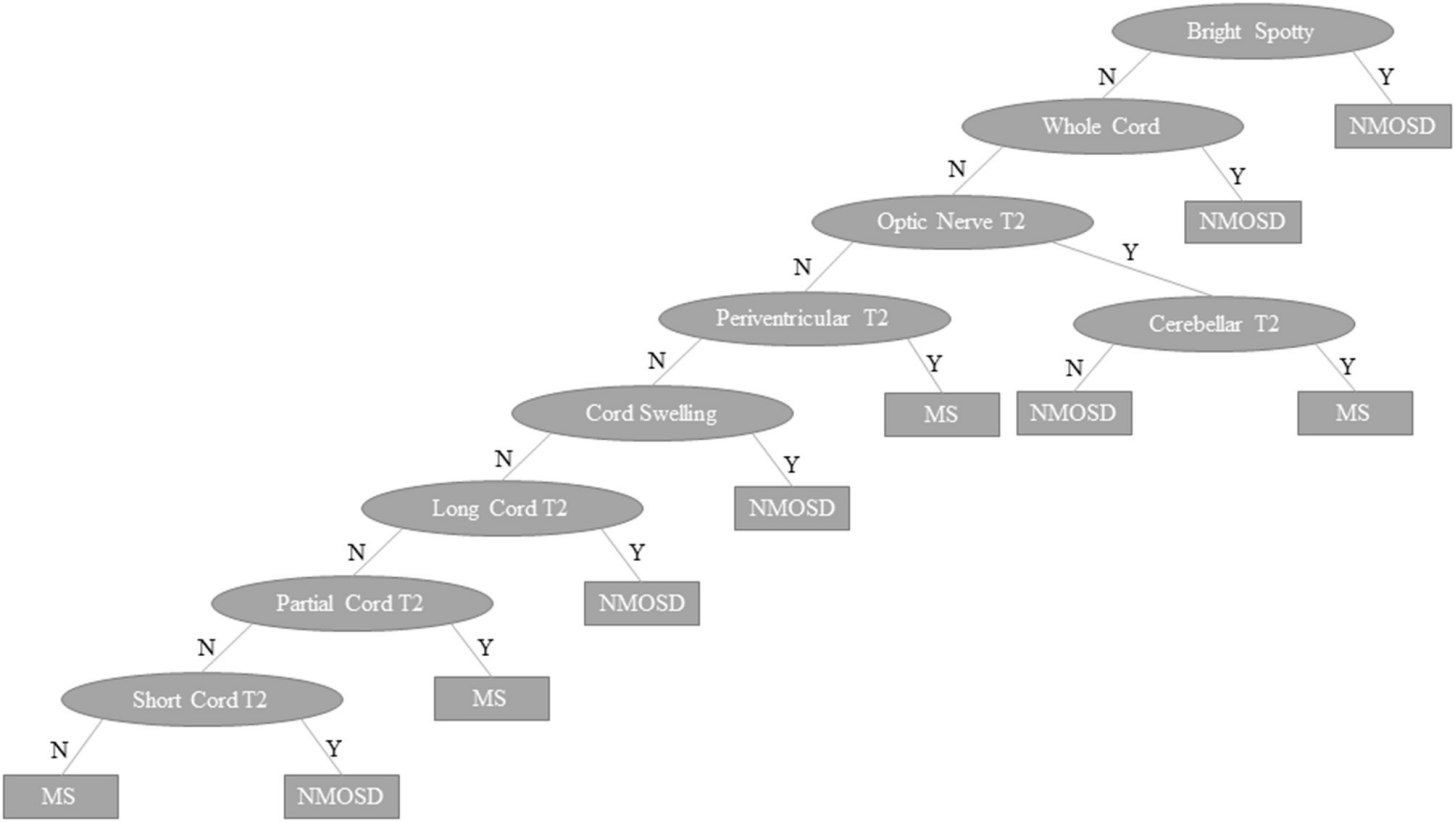
Machine learning decision tree to distinguish NMOSD from multiple sclerosis based binarised presence (Y) or absence (N) of specific lesion types and features as indicated in ovals. NMOSD, neuromyelitis optica spectrum disorder; MS, multiple sclerosis, Y, yes; N, no.

The outcome of the predictive modelling for NMOSD and MS based on MRI features seen on “ever” imaging is summarised in Supplementary Table 3. A total of 134 models were tested for NMOSD and 55 for MS but only the best models (models 1–4) are shown. For both MS and NMOSD predictive models, summative scores of features (see Supplementary Table 3) with weightings (for longitudinally extensive spinal cord and bilateral optic nerve lesions in NMOSD and ovoid lesions in MS) gave the best results. The NMOSD score gave a weighted mean precision of 0.921. Exclusion of spinal MRI data (model 5) reduced this to 0.802. A combined approach using both NMOSD/MS scores according to the rule, if the NMOSD score × 3.5 > MS score then the diagnosis is NMOSD and if not, the diagnosis is MS, gave an overall precision of 0.935. The machine learning decision tree is shown in Figure 11 and performed slightly better with precision of 0.939. The performance of these latter two models is shown in comparison to previous methods of predicting MS and NMOSD using the “first” MRI dataset in Table 4.

**Table 4:** Summary of predictive models for NMOSD and multiple sclerosis based on MRI.

					**Weighted Means**				
**Model (Ref)**	**TP**	**FP**	**TN**	**FN**	**TP Rate**	**FP Rate**	**Precision**	***F*-measure**	**ROC Area**
Paty (29)	29	9	91	33	0.637	0.259	0.752	0.669	0.689
Swanton (30)	48	43	57	14	0.696	0.352	0.633	0.642	0.672
Matthews/Juryńczyk/Hyun (14, 31, 33)	34	25	75	28	0.626	0.324	0.634	0.630	0.649
Liao (18)	5	0	100	57	0.432	4.352	0.861	0.390	0.540
Bensi (32)	33	24	76	29	0.619	0.327	0.634	0.626	0.646
NMO/MS score	53	9	91	9	0.876	0.111	0.876	0.876	0.882
Machine learning algorithm	52	9	91	10	0.866	0.117	0.871	0.868	0.874

*Criteria definitions*.

*Paty = 3 or more white matter lesions or if one periventricular lesion, 3 or more white matter lesions indicates MS, otherwise NMOSD*.

*Swanton = at least 2 of the following criteria: (1) periventricular lesion; (2) juxtacortical lesion; (3) infratentorial lesion; and (4) spinal cord lesion indicates MS, otherwise NMOSD*.

*Matthews = lesion adjacent to lateral body of ventricle (periventricular lesion) and inferior temporal lobe lesion, and U-fibre lesion (juxtracortical lesion) or Dawson's finger type lesion indicates MS, otherwise NMOSD*.

*Liao = linear ependymal lesion (pencil corpus callosum, hypothalamic, periaqueductal, periventricular, third ventricle) and not meeting Matthews criteria indicates NMSOD, otherwise MS*.

*NMOD/MS Score = NMOSD Score × 3.5 > MS score indicates NMSOD, otherwise MS*.

*NMOSD Score = counting 1 for each of Gd-enhancing, whole (axial) cord, swollen, central or atrophic lesion spinal cord lesions; Gd-enhancing, T2, chiasm or longitudinally extensive lesion optic nerve lesions; and nucleus tractus solitarius, periaqueductal, third ventricular, hypothalamic, leptomeningeal Gd-enhancing, central medullary, cloud-like or area postrema brain lesions, and counting 2 for longitudinally extensive spinal cord lesions and bilateral optic nerve lesions*.

*MS Score = counting 1 for each of short segment lesion and partial cord lesions, and ovoid, Dawson's fingers, pyramidal corpus callosum, other corpus callosum, periventricular, temporal lobe, splenium, cerebellar, large supratentorial, cortical, subcortical, juxtacortical, cerebellar peduncle T2 lesion, T1 black holes, brain Gd-enhancing lesion, and 9 or more T2 brain lesions*.

*Machine Learning Algorithm = see Figure 11 for algorithm*.

*Data is presented as ability to predict NMOSD vs. multiple sclerosis. Rates, precision and F-measure are all weighted average figures for predicting NMOSD and multiple sclerosis (i.e., overall accuracy)*.

*Ref, reference; TP, true positive; FP, false positive; TN, true negative; FN, false negative; ROC, receiver operator characteristic*.

In MS the Paty criteria (23) had a high sensitivity (0.910) but low specificity (0.468) for MS and was poor in predicting NMOSD. The Barkhof dissemination in space criteria (124) had higher specificity (0.774) in predicting MS with reasonable sensitivity (0.570). The previously published MRI criteria (14, 18, 32–34) for distinguishing NMOSD from MS performed well in identifying MS, but performed no better than the Barkhof criteria in identifying NMOSD. The NMOSD/MS score gave overall precision of 0.876 with the machine learning decision tree giving precision of 0.871. These models were able to accurately distinguish NMOSD from MS in 144/166 (87%) and 143/166 (86%) of cases, respectively, based on first available imaging alone. A sensitivity analysis restricted to first scans performed in the 5 years since onset of symptoms showed very similar results (precision for NMOSD/MS score = 0.900 and machine learning algorithm = 0.864).

## Discussion

We have undertaken a cross-sectional, comparison study of MRI data in AQP4 antibody positive NMOSD and MS cases analysing for typical lesions that have previously been described for each condition. We have compared how frequently these lesions are seen in the two disorders and have found patterns that are largely consistent with previously published reports (14, 18, 37, 42, 45, 49, 81). We note that NMOSD associated lesions in the brain were generally single lesions but collectively were seen in 40% of cases. Longitudinally extensive spinal cord lesions were seen in a majority of NMOSD cases (70%) at some point during their disease. Initially normal MRI brain (not meeting Paty criteria) was seen in 16% of NMOSD cases. The frequency of supratentorial T2 lesions, excepting the NMOSD specific lesions, was higher in MS compared to NMOSD. Gd-enhancing lesions of the spinal cord and optic nerve were more common in NMOSD and Gd-enhancing lesions in the brain were more common in MS. Analysis of lesion location in the spinal cord indicates that longitudinal, central cord lesions affecting the whole (axial) cord with swelling and Gd-enhancement are more common in NMOSD and short, partial cord lesions are more common in MS. The predictive value in distinguishing NMOSD from MS for these individual MRI features was limited but using scores for NMOSD and MS based on the occurrence of typical lesions for each or a machine learning decision tree proved useful in distinguishing the two conditions. When applied to the current dataset these criteria accurately predicted the correct diagnosis in over 85% of cases based on first available imaging alone and performed better than previously defined criteria (14, 18, 32–34). These findings need to be replicated in an independent cohort, but if proven to be accurate have the potential to be useful in directing appropriate further investigation with AQP4 antibody testing and in situations where AQP4 antibody testing is not available may provide some guidance with regards to treatment.

We did not see any anterior midbrain, PRES-like or Balo-like lesions in either NMOSD cases or MS cases. This probably reflects the relative rarity of these lesion types and conclusions about their frequency in the two disorders must await analysis in larger datasets. Similarly, leptomeningeal Gd-enhancement, chiasmal, central medullary and area postrema lesions were only seen in NMOSD, but given their low frequency, definitively excluding their occurrence in MS would require a dataset of several hundred if not thousands of cases. We were not able to confirm postulated associations with NMOSD for cystic brain, tumefactive, bridging splenium, brainstem periependymal, linear periventricular periependymal, heterogenous corpus callosum, pencil-like corpus callosum or cerebral peduncle lesions, which were seen with similar frequency in NMOSD and MS. This exemplifies the need to include large numbers of “control” MS comparison cases prior to defining lesions as being associated with NMOSD. These lesions appear to be more generically associated with demyelination of the central nervous system. Punctate and patch lesions have been noted as one of the most commonly seen brain lesions in NMOSD. In the present study these lesions were seen just as commonly in MS and with similar lesion counts within cases. We would note that these lesions are similar to the lesions described in migraine (125), a condition that is likely to be frequently seen in both NMOSD and MS as all three conditions are more common in women (126–128).

Two recent studies have confirmed the value of the Matthews criteria in distinguishing MS from NMOSD (34, 129). These studies found similar sensitivity for detecting MS at 79.8% (34) and 82.9% (129) compared to 75% in the present study and neither of these figures are as higher as the 91% sensitivity for diagnosing MS seen with the NMSOD/MS score method and the machine learning algorithm identified here. Our results accord well with a recent similar study comparing AQP4 antibody associated NMOSD and MS (130). We would suggest that a high MS score on MRI, in the absence of clinical features suggestive of NMOSD, AQP4 antibody testing is not indicated. Another study has looked specifically at corpus callosum lesions in NMOSD and MS, in common with the present study finding that overall corpus callosum lesions are seen equally as commonly in all regions (131). This study found that corpus callosum lesions in NMOSD were more likely to be diffuse, have blurred margins and be heterogeneous which is somewhat at odds with the data presented here. Similarly, “extensive” or bridging lesions of the splenium were seen more commonly in NMOSD but were also seen in MS (131) whilst these lesions seen equally in the two conditions in the present larger dataset. Others have also looked at the value of Brain MRI in distinguishing NMOSD and MOGAD from MS, finding lesion distribution is helpful (32).

Strengths of the present study include the comprehensive literature search for potentially associated MRI features, the large dataset of NMOSD and MS cases with near complete availability of brain and spine imaging, definition of NMOSD cases through highly specific assays for AQP4 antibodies, age and sex matching of MS cases, blinded assessment of scans and an expansive approach to predictive modelling to optimise the classification of the NMOSD and MS cases. Potential weaknesses of the study include the historical cohort nature of the MS cases leading to differences in disease duration and timing of first imaging. We have also not been able to test our predictive models in a truly independent dataset. However, the associated features used in the predictive models had all been previously identified as being associated with NMOSD or MS. The fact that more MRI in NMOSD were undertaken during a relapse may contribute to the greater frequency of Gd-enhancement seen in the optic nerve and spinal cord. Finally, the low frequency and difference between the two groups in dedicated orbital imaging, means that the findings in relation to optic nerve lesions may be underestimated. It should also be noted that these predictive models do not take into account the pre-test probability for a diagnosis of NMOSD, which in some regions of the world (e.g., Japan) may be as high as 50% (132) vs. perhaps 1% in populations of European ancestry (21).

In conclusion, the MRI features of NMOSD and MS are distinct and can be used to distinguish the two conditions with high precision early in the disease course. This makes our final predictive models of potential value in making initial management and treatment decisions, particularly in settings where AQP4 antibody testing is not readily available.

## Data Availability Statement

The raw data supporting the conclusions of this article will be made available to suitably qualified researchers subject to approval by the Griffith University HREC.

## Ethics Statement

This study was approved by Griffith University HREC. Written informed consent to participate in this research was provided by all participants or their legal guardian/next of kin.

## Author Contributions

This study was undertaken by the Australia and New Zealand Neuromyelitis Optica (ANZ NMO) Collaboration and all authors are members of this group. As such all authors took part in the design of this study and were involved in participant recruitment, data collection, laboratory testing of samples or MRI analysis. The primary data analysis was conducted by LC, SAB, and JSu. The group was coordinated by a group chaired by SAB, and consisted of AGKK, DM, MM, JDEP, MS, and BT. All authors have approved the final manuscript.

## Funding

This project was undertaken by the ANZ NMO Collaboration and was supported by funding from Multiple Sclerosis Research Australia (11-038), the Brain Foundation, Griffith University and the Gold Coast Hospital Foundation. The work in Oxford was supported by the National Health Service National Specialised Commissioning Group for Neuromyelitis Optica.

## Conflict of Interest

MHB has received research support, speaking engagement honoraria, advisory board honoraria and travel sponsorship from Biogen Idec, Merck, Novartis, Roche and Sanofi-Genzyme, and is a consulting neurologist for RxMx and is Research Director of the Sydney Neuroimaging Analysis Centre. MB has received travel sponsorship and honoraria from Sanofi-Genzyme, Teva, Novartis, Biogen Idec and Roche. BB has received honoraria as a board member for GlaxoSmithKline, Biogen Idec, ViiV Healthcare and Merck Serono, has received speaker honoraria from ViiV Healthcare, Boehringer Ingelheim, Abbott, Abbvie, and Biogen Idec; has received travel sponsorship from Abbott and ViiV Healthcare, and has received research support funding from EI Lilly, GlaxoSmithKline, ViiV Healthcare and Merck Serono. FB has received speaker's honoraria from Biogen-Idec and EMD Serono. SAB has received honoraria for attendance at advisory boards and travel sponsorship from Bayer-Schering, Biogen-Idec, Merck-Serono, Novartis, and Sanofi-Genzyme, has received speakers honoraria from Biogen-Idec and Genzyme, is an investigator in clinical trials sponsored by Biogen Idec, Novartis and Genzyme, and was the recipient of an unencumbered research grant from Biogen-Idec. HB has received honoraria for serving on scientific advisory boards for Biogen Idec, Novartis and Sanofi-Genzyme, has received conference travel sponsorship from Novartis and Biogen Idec, has received honoraria for speaking and acting as Chair at educational events organised by Novartis, Biogen Idec, Medscape and Merck Serono, serves on steering committees for trials conducted by Biogen Idec and Novartis, is chair (honorary) of the MSBase Foundation, which has received research support from Merck Serono, Novartis, Biogen Idec, Genzyme Sanofi and CSL Biopharma and has received research support form Merck Serono. WC has been the recipient of travel sponsorship from, and provided advice to, Bayer Schering Pharma, Biogen-Idec, Novartis, Genzyme, Sanofi-Aventis, BioCSL and Merck-Serono. RD has received research funding from the National Health and Medical Research Council, MS Research Australia, Star Scientific Foundation, Pfizer Neuroscience, Tourette Syndrome Association, University of Sydney, and the Petre Foundation and has received honoraria from Biogen-Idec and Bristol-Myers Squibb as an invited speaker. MF-P has received travel sponsorship from Biogen Australia and New Zealand. RH has received honoraria, educational support and clinic funding from Novartis, Biogen Idec, Genzyme and BioCSL. AGKK has received scientific consulting fees and/or lecture honoraria from Bayer, BioCSL, Biogen-Idec, Genzyme, Lgpharma, Merck, Mitsubishi Tanabe Pharma, NeuroScientific Biopharmaceuticals, Novartis, Roche, Sanofi-Aventis, and Teva. TK has received travel sponsorship from Novartis, BioCSL, Novartis, Merck Serono and Biogen Idec, has received speaker honoraria from Biogen Idec, BioCSL, Merck Serono, Teva, Genzyme and Novartis, has received research support from Biogen Idec, Genzyme, GlaxoSmithKline, Bayer-Schering and Merck Serono, and has received scientific consulting fees from GlaxoSmithKline China, Biogen-Idec and Novartis. JK has received remuneration for advisory board activities and presentations from Bayer Healthcare, Biogen Idec, BioCSL, Genzyme and Novartis. CK has received travel support, honoraria and advisory board payments from Biogen Idec, Bayer, Genzyme, Novartis and Serono. JL-S has received unencumbered funding as well as honoraria for presentations and membership on advisory boards from Sanofi Aventis, Biogen Idec, Bayer Health Care, CSL, Genzyme, Merck Serono, Novartis Australia and Teva. RM has received honoraria for attendance at advisory boards and travel sponsorship from Bayer-Schering, Biogen-Idec, CSL, Merck-Serono, Novartis, and Sanofi-Genzyme. MM has received travel sponsorship, honoraria, trial payments, research and clinical support from Bayer Schering, Biogen Idec, BioCSL, Genzyme, Novartis, and Sanofi Aventis Genzyme. DM has received honoraria for attendance at advisory boards from Biogen-Idec and Novartis, and travel sponsorship from Bayer-Schering, Biogen-Idec, and Sanofi-Genzyme. PM has received honoraria or travel sponsorship from Novartis, Sanofi-Aventis and Biogen Idec. JP has received travel sponsorship, honoraria for presentations and membership on advisory boards from Biogen Idec and Novartis and Sanofi Aventis. JDP has received honoraria for seminars or advisory boards from Teva, Biogen, Sanofi-Genzyme, Novartis, Merck, Bayer and research grants or fellowships from Merck, Novartis, Bayer, Biogen, Sanofi-Genzyme, and Teva. SR has received honoraria for advisory consultancy from UCB and speaker's honoraria from Biogen Idec. SWR has received travel sponsorship, honoraria, trial payments, research and clinical support from Aspreva, Baxter, Bayer Schering, Biogen Idec, BioCSL, Genzyme, Novartis, Sanofi Aventis Genzyme and Servier, and is a director of Medical Safety Systems Pty Ltd. CPS has received travel sponsorship from Biogen Idec, Novartis and Bayer-Schering. IS has received remuneration for Advisory Board activities from Biogen, CSL, and Bayer Schering and educational activities with Biogen, CSL and travel sponsorship from Biogen, Novartis and Bayer Schering. JMS has received honoraria for lectures and participation in advisory boards, and travel sponsorship from Novartis, BioCSL, Genzyme, and Biogen Idec. BT has received travel sponsorship from Novartis and Bayer Schering. AV and the University of Oxford hold patents and receive royalties for antibody testing. PW and the University of Oxford hold patents for antibody assays and have received royalties, has received honoraria from Alexion, Biogen Idec F. Hoffmann-La Roche, Retrogenix, UBC and Euroimmun AG, and travel grants from the Guthy-Jackson Charitable Foundation. EW has received honoraria for participation in advisory boards from Biogen-Idec and Novartis, travel sponsorship from Biogen-Idec, Bayer-Schering and Teva and is an investigator in clinical trials funded by Biogen-Idec and Teva. The remaining authors declare that the research was conducted in the absence of any commercial or financial relationships that could be construed as a potential conflict of interest.

## Publisher's Note

All claims expressed in this article are solely those of the authors and do not necessarily represent those of their affiliated organizations, or those of the publisher, the editors and the reviewers. Any product that may be evaluated in this article, or claim that may be made by its manufacturer, is not guaranteed or endorsed by the publisher.
