# Supplementary material for: MRI Patterns Distinguish AQP4 Antibody Positive Neuromyelitis Optica Spectrum Disorder From Multiple Sclerosis
Source: Front Neurol. 2021 Sep 9;12:722237. doi: 10.3389/fneur.2021.722237 (PMC8458658; doi:10.3389/fneur.2021.722237)
Supplement: Supplementary file 1 [file Table_1.pdf]

Supplementary Table 1. Results of literature search

Author/Year (Ref)	Found	Population	Study Type	Lesions Identified
Misu et al 2005 (37)	LS	NMOSD	Case series	Nucleus tractus solitarius in NMOSD
Nakashima et al 2006 (38)	LS	OSMS (AQP4 +ve vs -ve)	Case series	LESCL in NMOSD
Chan et al 2006 (39)	LS	ATM (NMOSD vs MS vs IDDD)	Case series	LESCL in NMOSD
Pittock et al 2006 (40)	LS	NMOSD	Case series	Brain abnormalities in NMOSD
Pittock et al 2006 (41)	LS	NMOSD	Case series	III ventricle; IV ventricle; hypothalamus; periaqueductal; central medulla; optic chiasm; superior cerebellar peduncle; subpial cerebellar in NMOSD
Scott et al 2006 (42)	LS	NMOSD vs MS	Comparison	Short cord; partial cord in MS
Cabrera-Gomez et al 2007 (43)	LS	NMOSD	Case series	Gd-enhancing ON in NMOSD
Bichuetti et al 2008 (44)	LS	NMOSD	Case series	Brain abnormalities in NMOSD
Cabrera-Gomez et al 2008 (45)	LS	NMOSD	Case series	Linear corpus callosum; linear periventricular; tumefactive; bridging splenium in NMOSD
Li et al 2008 (46)	LS	NMOSD	Case series	Punctate; patch; medulla in NMOSD
Nakamura et al 2008 (47)	LS	NMOSD	Case series	Central cord in NMOSD
Cassinotto et al 2009 (48)	LS	NMOSD	Case series	LESCL; central cord; Gd-enhancing cord; ring-enhancing cord; cord swelling in NMOSD
Ito et al 2009 (49)	LS	NMOSD	Case series	Cloud-like enhancement in NMOSD
Krampla et al 2009 (50)	LS	NMOSD	Case series	Central cord in NMOSD
Magana et al 2009 (51)	LS	NMOSD	Case series	PRES in NMOSD
Nakamura et al 2009 (52)	LS	NMOSD vs MS	Comparison	Large heterogeneous corpus callosum in NMOSD
Adoni et al 2010 (53)	LS	NMOSD	Case series	Whole (axial) cord; cervical and thoracic cord in NMOSD
Lu et al 2010 (54)	LS	NMOSD vs MS	Comparison	LESCL; central cord; linear cord; linear medulla in NMOSD
Matsushita et al 2010 (55)	LS	NMOSD vs MS	Comparison	Tumefactive in NMOSD: ovoid in MS

Chan et al 2011 (56)	LS	NMOSD	Case series	Periaqueductal; IV ventricle; central medulla; tumefactive; cloud-like in NMOSD
Ikeda et al 2011 (57)	LS	NMOSD	Case report	Tumefactive in NMOSD
Kim et al 2011 (58)	LS	NMOSD	Case series	Brain abnormalities in NMOSD
Li et al 2011 (59)	LS	ATM (AQP4 +ve vs -ve)	Case series	LESCL; central cord in NMOSD
Liu et al 2011 (60)	LS	NMOSD vs MS	Comparison	McDonald MRI criteria in MS
Lu et al 2011 (61)	LS	NMOSD vs MS vs ADEM	Comparison	Medulla; dorsal brainstem in NMSOD
Qiu et al 2011 (62)	LS	MS	Case series	Hypothalamic in MS
Wang et al 2011 (63)	LS	NMOSD	Case series	LESCL in NMOSD
Wang et al 2011 (64)	LS	NMOSD	Case series	Hypothalamic in NMOSD
Chen et al 2012 (65)	LS	NMOSD vs MS	Comparative	Periependymal ‘dot’ corpus callosum in NMOSD
Downer et al 2012 (16)	LS	NMOSD (AQP4 +ve vs -ve)	Case series	LESCL; central cord; cord T1 hypointensity; hypothalamic; periaqueductal in NMOSD
Khanna et al 2012 (66)	LS	NMOSD vs MS	Comparative	Optic chiasm in NMOSD
Lalan et al 2012 (67)	LS	NMOSD vs MS	Comparative	LESCL; central cord in NMOSD: short cord; peripheral cord in MS
Pires et al 2012 (68)	LS	NMOSD	Case series	Non-specific; patches; punctate; linear periventricular ependymal; IV ventricle in NMOSD
Zhong et al 2012 (69)	LS	NMOSD vs MS	Comparative	Central cord in NMOSD
Asgari et al 2013 (70)	LS	LETM (AQP4 +ve vs -ve)	Case series	Brainstem; hypothalamic; periaqueductal; central medullary and area postrema in NMSOD
Chan et al 2013 (71)	LS	NMOSD vs MS vs ADEM vs IDDD	Comparison	LESCL; normal brain in NMOSD
Iorio et al 2013 (72)	LS	LETM (AQP4 +ve vs -ve)	Case series	Central medulla; central cord in NMOSD
Kiyat-Atamer et al 2013 (73)	LS	NMOSD (AQP4 +ve vs -ve)	Case series	LESCL in NMSOD
Makino et al 2013 (74)	LS	NMOSD vs MS	Comparative	Heterogenous splenium; bridging splenium in NMOSD
Matthews et al 2013 (14)	LS	NMOSD vs MS	Comparative	Periventricular; inferior temporal; juxtacortical; Dawson’s fingers in MS: Tumefactive; thalamic; cerebral peduncle; IV ventricle in NMOSD

Barhate et al 2014 (75)	LS	NMOSD	Case series	LESCL; central medulla in NMOSD
Berger et al 2014 (76)	LS	NMOSD	Case report	PRES in NMOSD
Huh et al 2014 (77)	LS	NMOSD vs MS	Comparison	Ovoid; juxtacortical; perpendicular periventricular in MS: Longitudinal CST; tumefactive; linear periventricular periependymal; central medullar in NMOSD
Kim et al 2014 (78)	LS	NMOSD	Case series	Cystic in NMOSD
Kulkarni et al 2014 (79)	LS	NMOSD	Case report	Area postrema in NMOSD
Liao et al 2014 (18)	LS	NMOSD vs MS	Comparison	Pyramidal corpus callosum; brainstem ependymal dot in MS: Punctate; linear ependymal in NMOSD
Lim et al 2014 (80)	LS	ON (NMOSD vs MS)	Comparison	Chiasm in NMOSD
Lin et al 2014 (81)	LS	NMOSD	Case series	LESCL; cervical and thoracic cord in NMOSD
Liu et al 2014 (82)	LS	NMOSD vs MS	Comparison	LESCL in NMOSD: brainstem in MS
Long et al 2014 (83)	LS	NMOSD vs MS	Comparison	‘Cloud-like’; ‘pencil thin’ periependymal; periventricular enhancement; IV ventricle enhancement; leptomeningeal enhancement in NMOSD: white matter enhancement in MS
Yonezu et al 2014 (84)	LS	NMOSD vs MS	Comparison	‘Bright spotty’ cord in NMOSD
Zhang et al 2014 (85)	LS	NMOSD vs MS vs ADEM	Comparison	Hypothalamus in NMOSD: Thalamus equivalent in NMOSD and MS
Absoud et al 2015 (86)	LS	Paediatric NMOSD	Case series	Abnormal brain in NMOSD
Eshaghi et al 2015 (87)	LS	NMOSD vs MS vs Healthy	Comparison	T2 lesion volume in MS
Flanagan et al 2015 (88)	LS	TM (AQP4 +ve vs -ve)	Comparison	Central cord; T1 hypointense cord; LESCL in NMOSD; Barkhof criteria in MS
Igel et al 2015 (89)	LS	NMOSD	Case report	PRES in NMOSD
Lemos et al 2015 (90)	LS	NMOSD	Case series	LESCL; abnormal brain in NMOSD
Mealy et al 2015 (91)	LS	NMOSD vs MS	Comparison	Long ON in NMOSD
Wingerchuk 2015 (1)	LS	NMOSD	Diagnostic Criteria	Normal MRI brain; long ON lesion; optic chiasm; LESCL; area postrema; periependymal brainstem in NMOSD
Yokote et al 2015 (92)	LS	NMOSD	Case series	Ring-enhancing cord in NMOSD

Kim et al 2016 (93)	LS	NMOSD	Case series	Heterogeneous corpus callosum; longitudinal CST; cerebral peduncle; IV ventricle; tumefactive; linear periventricular ependymal; hypothalamic in NMOSD
Kim et al 2016 (94)	LS	NMOSD	Case series	Leptomeningeal enhancement in NMSOD
Kister et al 2016 (95)	LS	TM (NMOSD vs MS)	Comparison	High cervical cord; extension to pial surface cord; 'bright spotty' cord; enhancing cord in NMOSD
Flanagan et al 2016 (96)	LS	NMOSD vs Sarcoid	Comparison	Ring-enhancement of cord in NMOSD
Pekcevik et al 2016 (97)	LS	LETM	Case series	'Bright spotty' cord; central cord; whole (axial) cord in NMOSD
Abdullah et al 2017 (98)	LS	AQP4 +ve vs IDDD	Comparison	Fewer lesions in NMOSD: McDonald MRI criteria in IDDD
Buch et al 2017 (99)	LS	ON (NMOSD vs MS)	Comparison	Long ON; bilateral ON; chiasm in MMOSD: McDonald criteria in MS
Dumrikarnlert et al 2017 (100)	LS	TM (NMOSD vs MS vs IDDD)	Comparison	Cervical cord; partial cord in MS: LESCL; central; cervico-thoracic in NMOSD
Hayashida et al 2017 (101)	LS	NMOSD	Case series	Central cord, whole cord in NMOSD
Huh et al 2017 (102)	LS	NMOSD (AQP4 +ve)	Case series	Short cord in NMOSD
Jurynczyk et al 2017 (32)	LS	NMOSD vs MS vs MARD	Comparison	Matthews criteria in MS
Zalewski et al 2017 (103)	LS	NMOSD vs MS	Comparison	Ring-enhancing cord in NMOSD
Bensi et al 2018 (33)	LS	NMOSD vs MOGAD vs MS	Comparison	Periventricular, inferior temporal lobe, juxtacortical, Dawson finger, short segment spinal cord lesion in MS
Carnero Contentti et al 2018 (104)	LS	NMOSD	Case series	Optic chiasm, corticospinal tract, third ventricle, thalamus, hypothalamus, area postrema in NMSOD
Chee et al 2018 (105)	LS	NMSOD	Case series	Central medullary, cord swelling, bright spotty, central cord in NMSOD
Hu et al 2018 (106)	LS	NMOSD	Case series	LESCL in NMOSD
Lu et al 2018 (107)	LS	NMOSD vs MS	Comparison	Optic chiasm in NMOSD
Mehdipour-Dastjerdi et al 2018 (108)	LS	NMOSD	Case series	Thalamic, hypothalamic, III ventricle, IV ventricle, are postrema, linear periependymal periventricular, LESCL, short segment spinal cord in NMOSD
Tatekawa et al 2018 (109)	LS	NMOSD vs MS	Comparison	Spinal cord atrophy, spinal cord swelling, central cord in NMOSD; ovoid, T1 black hole, callosal-septal-interface, juxtacortical in MS
Hyun et al 2019 (34)	LS	NMOSD vs MOGAD vs MS	Comparison	Periventricular, inferior temporal lobe, juxtacortical, Dawson finger in MS

Kim et al 2010 (17)	B/P	NMOSD	Case series	Longitudinal CST; tumefactive; III ventricle; IV ventricle; linear periventricular ependymal; central medullary; non-specific brain; LESCL in NMOSD
Matsuoka et al 2010 (110)	B/P	NMOSD	Case report	Balo's in NMOSD
Banker et al 2012 (111)	B/P	NMOSD	Case series	Ependymal Gd; linear periventricular ependymal in NMOSD
Pula et al 2014 (112)	B/P	NMOSD vs MS vs Sarcoid	Comparison	Long ON in NMOSD
Cai et al 2016 (113)	B/P	NMOSD	Case series	LESCL; linear spinal cord in NMOSD
Fazekas et al 1988 (114)	B/P	MS vs Healthy	Comparison	Large (> 6mm); periventricular; infratentorial in MS
Paty et al 1988 (23)	B/P	MS vs CIS	Comparison	Paty criteria in MS
Barkhof et al 1997 (24)	B/P	MS vs CIS	Comparison	Gd-enhancing brain; juxtacortical; periventricular; infratentorial in MS
Bakshi et al 1998 (115)	B/P	TM vs MS	Comparison	Short segment cord, partial spinal cord in MS
Tintore et al 2000 (116)	B/P	MS vs CIS	Case series	Paty; Fazekas; Barkhof in MS
Korteweg et al 2006 (117)	B/P	MS vs CIS	Comparison	Barkhof/Tintore criteria in MS
Tintore et al 2006 (118)	B/P	MS vs CIS	Comparison	Barkhof; >9 lesions in MS
Swanton et al 2007 (31)	B/P	MS vs CIS	Comparison	Juxtacortical; periventricular; infratentorial; spinal cord; new T2 in MS
Polman et al 2011 (19)	B/P	MS	Diagnostic Criteria	Periventricular; juxtacortical; infratentorial; spinal cord; new T2; new Gd in MS
Ruet et al 2011 (119)	B/P	TM vs MS	Comparison	>1 spinal cord; partial spinal cord; >8 brain; >2 periventricular in MS
Bourre et al 2012 (120)	B/P	TM vs MS	Comparison	Abnormal MR brain; Barkhof criteria; partial spinal cord; Gd spinal cord in MS

LS = literature search; B/P = bibliographic search or personal knowledge; ADEM = acute disseminated encephalomyelitis; IDDD = idiopathic disseminated demyelinating disease; MS = multiple sclerosis; NMOSD = neuromyelitis optica spectrum disorder; AQP4 = aquaporin 4 antibody;+ve = positive; -ve = negative; ON = optic neuritis; ATM = acute transverse myelitis; LETM = longitudinally extensive transverse myelitis; TM = transverse myelitis; LESCL = longitudinally extensive spinal cord lesion; CST = corticospinal tract; CIS = clinically isolated syndrome; PRES = posterior reversible encephalopathy syndrome.
