# Supplementary material for: MRI Patterns Distinguish AQP4 Antibody Positive Neuromyelitis Optica Spectrum Disorder From Multiple Sclerosis
Source: Front Neurol. 2021 Sep 9;12:722237. doi: 10.3389/fneur.2021.722237 (PMC8458658; doi:10.3389/fneur.2021.722237)
Supplement: Supplementary file 2 [file Table_2.pdf]

Supplementary Table 2. Frequencies, sensitivity, specificity and odds ratios for lesions in NMOSD and MS

Lesion	NMOSD			MS			OR (95% CI)
	Frequency n/N (%)	Sensitivity	Specificity	Frequency n/N (%)	Sensitivity	Specificity	
**Longitudinally Extensive Spinal Cord T2 Lesion (NMOSD)**	**43/61 (70)**	**0.705**	**0.988**	**1/86 (1)**	**0.012**	**0.295**	**203.056 (26.223 - 1572.34)**
**Bright Spotty Spinal Cord T2 Lesion (NMOSD)**	**32/61 (52)**	**0.525**	**0.988**	**1/86 (1)**	**0.012**	**0.475**	**93.793 (12.263 - 717.392)**
**Whole (Axial) Spinal Cord T2 Lesion (NMOSD)**	**15/61 (25)**	**0.250**	**0.994**	**0/86 (0)**	**0.006**	**0.750**	**57.667 (3.374 - 985.702)**
**Bilateral Optic Nerve T2 or Gd-enhancing T1 Lesions (NMOSD)**	**8/62 (13)**	**0.135**	**0.995**	**0/100 (0)**	**0.005**	**0.865**	**31.349 (1.775 - 553.567)**
**Gd-enhancing Spinal Cord T1 Lesion**	**17/55 (31)**	**0.309**	**0.985**	**1/65 (2)**	**0.015**	**0.691**	**28.632 (3.662 - 223.831)**
**Nucleus Tractus Solitarius T2 Lesion (NMOSD)**	**5/62 (9)**	**0.087**	**0.995**	**0/100 (0)**	**0.005**	**0.913**	**19.226 (1.044 - 354.059)**
Leptomeningeal Gd-enhancing T1 Lesion (NMOSD)	4/52 (8)	0.085	0.995	0/100 (0)	0.005	0.915	18.649 (0.984 - 353.414)
**Periaqueductal T2 Lesion (NMOSD)**	**9/62 (15)**	**0.145**	**0.990**	**1/100 (1)**	**0.010**	**0.855**	**16.811 (2.074 - 136.297)**
Optic Chiasm T2 or Gd-enhancing T1 Lesion (NMOSD)	4/62 (7)	0.071	0.995	0/100 (0)	0.005	0.929	15.462 (0.818 - 292.328)
Central Medullary T2 Lesion (NMOSD)	4/62 (7)	0.071	0.995	0/100 (0)	0.005	0.929	15.462 (0.818 - 292.328)
**Optic Nerve Gd-enhancing T1 Lesion**	**7/52 (13)**	**0.135**	**0.990**	**1/100 (1)**	**0.010**	**0.865**	**15.4 (1.84 - 128.911)**
**Third Ventricular Periependymal T2 Lesion (NMOSD)**	**7/62 (11)**	**0.113**	**0.990**	**1/100 (1)**	**0.010**	**0.887**	**12.6 (1.511 - 105.088)**
**Spinal Cord Swelling (NMOSD)**	**13/61 (21)**	**0.213**	**0.977**	**2/86 (2)**	**0.023**	**0.787**	**11.375 (2.462 - 52.552)**
Cloud-like Gd-enhancing T1 Lesion (NMOSD)	2/52 (5)	0.047	0.995	0/100 (0)	0.005	0.953	9.95 (0.469 - 211.2)
**Central Spinal Cord T2 Lesion (NMOSD)**	**43/58 (74)**	**0.741**	**0.772**	**18/79 (23)**	**0.228**	**0.259**	**9.715 (4.415 - 21.375)**
**Optic Nerve T2 Lesion**	**20/62 (32)**	**0.323**	**0.950**	**5/100 (5)**	**0.050**	**0.677**	**9.048 (3.182 - 25.729)**
Area Postrema T2 Lesion (NMOSD)	2/62 (4)	0.040	0.995	0/100 (0)	0.005	0.960	8.306 (0.392 - 175.928)

**Hypothalamic T2 Lesion (NMOSD)**	**8/62 (13)**	**0.129**	**0.980**	**2/100 (2)**	**0.020**	**0.871**	**7.259 (1.488 - 35.41)**
**Initial Brain MRI Normal (NMOSD)**	**10/62 (16)**	**0.161**	**0.970**	**3/100 (3)**	**0.030**	**0.839**	**6.218 (1.639 - 23.592)**
**Spinal Cord Atrophy (NMOSD)**	**12/61 (20)**	**0.197**	**0.953**	**4/86 (5)**	**0.047**	**0.803**	**5.02 (1.534 - 16.431)**
Longitudinal Corticospinal Tract T2 Lesion (NMOSD)	1/62 (2)	0.024	0.995	0/100 (0)	0.005	0.976	4.902 (0.197 - 122.253)
Longitudinal T2 Optic Nerve Lesion (NMOSD)	1/62 (2)	0.024	0.995	0/100 (0)	0.005	0.976	4.902 (0.197 - 122.253)
Cystic Brain Lesion (NMOSD)	3/62 (5)	0.048	0.980	2/100 (2)	0.020	0.952	2.492 (0.404 - 15.349)
Cerebellar Gd-enhancing T1 Lesion	1/52 (2)	0.019	0.990	1/100 (1)	0.010	0.981	1.941 (0.119 - 31.679)
Patch White Matter T2 Lesion (NMOSD)	33/62 (53)	0.532	0.620	38/100 (38)	0.380	0.468	1.857 (0.977 - 3.527)
Tumefactive Brain T2 Lesion (NMOSD)	1/62 (2)	0.016	0.990	1/100 (1)	0.010	0.984	1.623 (0.1 - 26.427)
Subcortical Gd-enhancing T1 Lesion	3/52 (6)	0.058	0.950	5/100 (5)	0.050	0.942	1.163 (0.267 - 5.071)
Bridging Splenium T2 Lesion (NMOSD)	2/62 (3)	0.032	0.970	3/100 (3)	0.030	0.968	1.078 (0.175 - 6.638)
Brainstem Periependymal T2 Lesion (NMOSD)	2/62 (3)	0.032	0.970	3/100 (3)	0.030	0.968	1.078 (0.175 - 6.638)
Linear Periventricular Periependymal T2 Lesion (NMOSD)	8/62 (13)	0.129	0.860	14/100 (14)	0.140	0.871	0.91 (0.358 - 2.313)
Brainstem T2 Lesion (MS)	13/62 (21)	0.210	0.770	23/100 (23)	0.230	0.790	0.888 (0.412 - 1.916)
Rounded Corpus Callosum T2 Lesion	1/62 (2)	0.016	0.980	2/100 (2)	0.020	0.984	0.803 (0.071 - 9.049)
Heterogeneous Corpus Callosum T2 Lesion (NMOSD)	3/62 (5)	0.048	0.940	6/100 (6)	0.060	0.952	0.797 (0.192 - 3.308)
Large Infratentorial T2 Lesion	8/62 (13)	0.129	0.840	16/100 (16)	0.160	0.871	0.778 (0.312 - 1.942)
Pencil-like Callosum T2 Lesion (NMOSD)	8/62 (13)	0.129	0.840	16/100 (16)	0.160	0.871	0.778 (0.312 - 1.942)
Punctate White Matter T2 Lesion (NMOSD)	10/62 (16)	0.161	0.800	20/100 (20)	0.200	0.839	0.769 (0.334 - 1.774)
Cerebral Peduncle T2 Lesion (NMOSD)	8/62 (13)	0.129	0.830	17/100 (17)	0.170	0.871	0.723 (0.292 - 1.792)
Thalamic T2 Lesion	2/62 (3)	0.032	0.950	5/100 (5)	0.050	0.968	0.633 (0.119 - 3.369)

Cerebellar Peduncle Gd-enhancing T1 Lesion	0/52 (0)	0.009	0.985	1/100 (1)	0.015	0.991	0.632 (0.025 - 15.781)
Corpus Callosum Gd-enhancing T1 Lesion	0/52 (0)	0.009	0.985	1/100 (1)	0.015	0.991	0.632 (0.025 - 15.781)
Deep Grey Matter T2 Lesion	7/62 (11)	0.113	0.810	19/100 (19)	0.190	0.887	0.543 (0.214 - 1.378)
**Partial Spinal Cord T2 Lesion (MS)**	**29/58 (50)**	**0.500**	**0.316**	**54/79 (68)**	**0.684**	**0.500**	**0.463 (0.23 - 0.932)**
Cerebellar Peduncle T2 Lesion (MS)	8/62 (13)	0.129	0.740	26/100 (26)	0.260	0.871	0.422 (0.177 - 1.003)
**Short Spinal Cord T2 Lesion (MS)**	**29/61 (48)**	**0.475**	**0.291**	**61/86 (71)**	**0.709**	**0.525**	**0.371 (0.187 - 0.737)**
**Juxtacortical T2 Lesion (MS)**	**35/62 (56)**	**0.565**	**0.220**	**78/100 (78)**	**0.780**	**0.435**	**0.366 (0.183 - 0.729)**
**Subcortical T2 Lesion**	**37/62 (60)**	**0.597**	**0.190**	**81/100 (81)**	**0.810**	**0.403**	**0.347 (0.17 - 0.708)**
**Cortical T2 Lesion**	**15/62 (24)**	**0.242**	**0.510**	**49/100 (49)**	**0.490**	**0.758**	**0.332 (0.165 - 0.67)**
Any Brain Gd-enhancing T1 Lesion	3/52 (6)	0.058	0.830	17/100 (17)	0.170	0.942	0.299 (0.083 - 1.072)
**Large T2 Brain Lesion (MS)**	**27/62 (44)**	**0.435**	**0.270**	**73/100 (73)**	**0.730**	**0.565**	**0.285 (0.146 - 0.557)**
**Large Supratentorial T2 Lesion**	**24/62 (39)**	**0.387**	**0.290**	**71/100 (71)**	**0.710**	**0.613**	**0.258 (0.132 - 0.504)**
**Cerebellar T2 Lesion (MS)**	**4/62 (6)**	**0.065**	**0.760**	**24/100 (24)**	**0.240**	**0.935**	**0.218 (0.072 - 0.664)**
**Initial MRI Brain Meets Barkhof Criteria (MS)**	**15/62 (24)**	**0.242**	**0.400**	**60/100 (60)**	**0.600**	**0.758**	**0.213 (0.105 - 0.431)**
**Inferior Temporal Lobe T2 Lesion (MS)**	**6/62 (10)**	**0.097**	**0.650**	**35/100 (35)**	**0.350**	**0.903**	**0.199 (0.078 - 0.508)**
**New Brain T2 Lesion (MS)**	**13/62 (21)**	**0.210**	**0.420**	**58/100 (58)**	**0.580**	**0.790**	**0.192 (0.093 - 0.398)**
**Splenium T2 Lesion**	**2/62 (3)**	**0.032**	**0.850**	**15/100 (15)**	**0.150**	**0.968**	**0.189 (0.042 - 0.857)**
**Any Corpus Callosum T2 Lesion**	**8/62 (13)**	**0.129**	**0.550**	**45/100 (45)**	**0.450**	**0.871**	**0.181 (0.078 - 0.42)**
**Hypodense T1 Brain Lesion (Black Hole)**	**6/62 (10)**	**0.097**	**0.590**	**41/100 (41)**	**0.410**	**0.903**	**0.154 (0.061 - 0.391)**
New Brain Gd-enhancing T1 Lesion (MS)	0/52 (0)	0.009	0.936	6/100 (6)	0.064	0.991	0.138 (0.008 - 2.507)
**Any Temporal Lobe T2 Lesion**	**8/62 (13)**	**0.129**	**0.480**	**52/100 (52)**	**0.520**	**0.871**	**0.137 (0.059 - 0.317)**

**Periventricular T2 Lesion (MS)**	**27/62 (44)**	**0.435**	**0.150**	**85/100 (85)**	**0.850**	**0.565**	**0.136 (0.065 - 0.286)**
**Initial MRI Brain Meets Paty Criteria (MS)**	**37/62 (60)**	**0.597**	**0.080**	**92/100 (92)**	**0.920**	**0.403**	**0.129 (0.053 - 0.311)**
Juxtacortical Gd-enhancing T1 Lesion	0/52 (0)	0.009	0.926	7/100 (7)	0.074	0.991	0.119 (0.007 - 2.121)
**Other Corpus Callosum T2 Lesion**	**1/62 (2)**	**0.016**	**0.860**	**14/100 (14)**	**0.140**	**0.984**	**0.101 (0.013 - 0.786)**
Periventricular Gd-enhancing T1 Lesion	0/52 (0)	0.009	0.896	10/100 (10)	0.104	0.991	0.082 (0.005 - 1.43)
**Pyramidal Corpus Callosum T2 Lesion (MS)**	**1/62 (2)**	**0.016**	**0.780**	**22/100 (22)**	**0.220**	**0.984**	**0.058 (0.008 - 0.443)**
**Dawson's Finger T2 Lesion (MS)**	**0/62 (0)**	**0.008**	**0.797**	**20/100 (20)**	**0.203**	**0.992**	**0.031 (0.002 - 0.53)**
**Ovoid T2 Lesion (MS)**	**1/62 (2)**	**0.016**	**0.640**	**36/100 (36)**	**0.360**	**0.984**	**0.029 (0.004 - 0.219)**

Entries in **bold** were significantly different

NMOSD = neuromyelitis optica; MS = multiple sclerosis; Gd = gadolinium, OR = odds ratios; CI = confidence interva
