# Supplementary material for: MRI Patterns Distinguish AQP4 Antibody Positive Neuromyelitis Optica Spectrum Disorder From Multiple Sclerosis
Source: Front Neurol. 2021 Sep 9;12:722237. doi: 10.3389/fneur.2021.722237 (PMC8458658; doi:10.3389/fneur.2021.722237)
Supplement: Supplementary file 3 [file Table_3.pdf]

Supplementary Table 3. MRI predictive models in distinguishing NMOSD from MS

					Weighted Means				
Model	TP	FP	TN	FN	TP Rate	FP Rate	Precision	F-measure	ROC Area
1	55	45	55	11	0.721	0.337	0.663	0.663	0.692
2	60	37	63	6	0.798	0.259	0.736	0.740	0.770
3	42	7	93	24	0.753	0.187	0.832	0.781	0.783
4	60	6	94	6	0.921	0.072	0.921	0.921	0.925
5	46	12	88	20	0.770	0.193	0.802	0.783	0.788
6	63	6	94	3	0.949	0.054	0.935	0.942	0.947
7	60	4	96	6	0.929	0.060	0.939	0.934	0.935

Frequencies are for predicting NMOSD (positive state) versus MS (negative state).

TP = true positive, FP = false positive, TN = true negative, FN = false negative, ROC = received operator characteristic

Models

1 = Best Regional Model to predict MS – 1 or more "MS spinal features" (short segment lesion or partial cord lesion) AND 4 or more "MS brain features" (ovoid, Dawson's fingers, pyramidal corpus callosum, other corpus callosum, periventricular, temporal lobe, splenium, cerebellar, large supratentorial, cortical, subcortical, juxtacortical or cerebellar peduncle lesion or T1 black holes or brain Gd-enhancing lesion, or 9 or more T2 brain lesions)

2 = Best Summative Model to predict MS ("MS Score")– scoring 8 or more when summing all MS spinal and brain features as listed in 1 above, counting each feature as 1 for present and 0 for not present, but weighting ovoid lesion as 2.

3 = Best Regional Model to predict NMOSD – 2 or more regions with at least 1 "NMOSD feature" as follows: spinal cord – longitudinally extensive, Gd-enhancing, whole (axial) cord, swollen, central or atrophic lesion; optic nerve – bilateral, Gd-enhancing, T2, chiasm or longitudinally extensive lesion; and brain – nucleus tractus solitarius, periaqueductal, third ventricular, hypothalamic, leptomeningeal Gd-enhancing, central medullary, cloud-like or area postrema lesion.

4 = Best Summative Model to predict NMOSD ("NMOSD Score") – scoring 3 or more when summing all "NMOSD features" from all three regions (spinal cord, optic nerve and brain) as listed in 3 above, counting each feature as 1 for present and 0 for not present, but weighting longitudinally extensive spinal cord lesions and bilateral optic nerve lesions as 2.

5 = Best Summative Model to predict NMOSD without spinal cord MRI – as for 4 above but not including "NMOSD spinal cord features".

6 = Best Composite Summative Model combining Best Summative Models for NMOSD and MS – both scores combined as follows: IF NMOSD score x 3.5 > MS score THEN "NMOSD" ELSE "MS"

7 = Machine Learning Model – see Figure 11 for algorithm.
